# Leveraging cross-source heterogeneity to improve the performance of bulk gene expression deconvolution

**DOI:** 10.1101/2024.04.07.588458

**Published:** 2024-04-09

**Authors:** Wenjun Shen, Cheng Liu, Yunfei Hu, Yuanfang Lei, Hau-San Wong, Si Wu, Xin Maizie Zhou

**Affiliations:** 1 Department of Bioinformatics, Shantou University Medical College, Shantou, Guangdong 515041, China; 2 Department of Computer Science, Shantou University, Shantou, Guangdong 515041, China; 3 Department of Computer Science, Vanderbilt University, Nashville, TN 37235, USA; 4 Department of Computer Sciences, City University of Hong Kong, Kowloon, Hong Kong; 5 Department of Computer Science, South China University of Technology, Guangzhou, Guangdong 510006, China; 6 Department of Biomedical Engineering, Vanderbilt University, Nashville, TN 37235, USA

## Abstract

A main limitation of bulk transcriptomic technologies is that individual measurements normally contain contributions from multiple cell populations, impeding the identification of cellular heterogeneity within diseased tissues. To extract cellular insights from existing large cohorts of bulk transcriptomic data, we present CSsingle, a novel method designed to accurately deconvolve bulk data into a predefined set of cell types using a scRNA-seq reference. Through comprehensive benchmark evaluations and analyses using diverse real data sets, we reveal the systematic bias inherent in existing methods, stemming from differences in cell size or library size. Our extensive experiments demonstrate that CSsingle exhibits superior accuracy and robustness compared to leading methods, particularly when dealing with bulk mixtures originating from cell types of markedly different cell sizes, as well as when handling bulk and single-cell reference data obtained from diverse sources. Our work provides an efficient and robust methodology for the integrated analysis of bulk and scRNA-seq data, facilitating various biological and clinical studies.

## 1 Introduction

Consideration of cellular heterogeneity is crucial when investigating the microenvironment of a disease-relevant tissue, as it plays a crucial role in identifying specific cellular populations that are potential therapeutic targets [1,2]. The advent of single-cell RNA sequencing (scRNA-seq) technologies has revolutionized the study of cellular heterogeneity by offering unprecedented resolution and genome-wide range in diverse diseased tissues [3–5]. However, the high cost and requirements for high-quality tissues impede the utilization of this approach in clinical investigations that typically involve a considerable cohort of participants [6,7].

Conventional gene expression profiling technologies, such as microarrays or bulk RNA-sequencing (RNA-seq) have successfully measured an enormous number of bulk samples due to technical simplicity and low cost. However, bulk RNA-seq technologies, which measure averaged expression levels in cell populations, hinder the elucidation of precise molecular signatures that govern tumor initiation and progression, as well as the identification of therapeutic targets specific to rare cell types. Over the last two decades, numerous computational methods have been developed to infer cell type composition from bulk gene expression data, commonly known as cell type deconvolution. This provides a cost-effective way to investigate the cellular heterogeneity in large cohorts of samples [8,9]. Two essential components are critical for accurate estimation of cell type composition from bulk data: (i) a specialized cell type-specific gene expression profile (GEP) matrix, often referred to as reference matrix or signature matrix, typically derived from scRNA-seq data and (ii) the ability to accommodate technical (e.g. discrepancies generated by different sequencing methods) and biological variations (e.g. differences in disease conditions) between the bulk and reference data. To build a signature matrix, a number of existing deconvolution methods [10–14] have utilized single-cell gene expression matrices measured by raw gene counts, which can be either read counts or UMI (Unique Molecular Identifier) counts [15]. This kind of method does not adequately address the significant impact of sequencing depth (total number of reads or UMI molecules per cell) on gene expression measurement. When the observed sequencing depth varies significantly between cells in the reference data, the resulting estimates of cell type proportions may not be accurate. On the other hand, to account for variable sequencing depth, many deconvolution methods have used single-cell GEPs measured by transcripts per million (TPM) or counts per million (CPM)-normalized values [16–20]. However, both TPM and CPM-based methods were designed to mitigate the effects of library size heterogeneity by employing count depth scaling, presuming that all cells in the data set initially possess an equivalent quantity of mRNA molecules [21]. When employing such a signature matrix to deconvolve bulk mixtures consisting of cell types with markedly different cell sizes, the resulting estimates of cell type proportions may lack accuracy without considering cell size factors. To our knowledge, the impact of cell size difference on deconvolution accuracy has not been systematically evaluated.

The majority of existing deconvolution methods primarily concentrate on addressing the influence of reference profiles and gene feature selection. Specifically, Cibersort [16] identifies gene features by measuring signature matrix stability via a 2-norm condition number. Methods, such as MuSiC [10], SCDC [12] and MuSiC2 [14], construct a reference matrix using multi-subject scRNA-seq data and utilizes a weighted non-negative least squares (NNLS) regression framework to mitigate bias in subject selection in scRNA-seq reference. DWLS [11] introduces a dampened weighted least squares regression framework by up-weighting the influence of lowly expressed marker genes. Empirical evidence indicates that these gene feature selection or weighting strategies result in a high deconvolution accuracy. However, only a few studies have focused on addressing the technical and biological disparities between the bulk and reference data. Bisque [20] learns gene-specific transformations of the bulk data to accommodate technical biases between the bulk and reference expression profiles. CIBERSORTx [19], an extension of CIBERSORT [16], designs a batch correction approach to mitigate cross-platform variation between the bulk mixtures and signature matrix. Nevertheless, CIBERSORTx requires a minimum of three bulk mixtures and recommends at least ten to perform the batch correction procedure. To the best of our knowledge, no deconvolution method currently exists that adequately and efficiently addresses the technical and biological variations between a single bulk mixture and the signature matrix.

To address these prevailing challenges, we introduce CSsingle (Cross-Source SINGLE cell deconvolution), an innovative cross-source deconvolution method that accurately and efficiently estimates cell type composition for both bulk and single-cell reference data from different sources. By explicitly addressing biological and technical variations inherent in individual bulk mixtures and the signature matrix, CSsingle significantly outperforms current state-of-the-art methods in precisely estimating cell type proportions for deconvolution tasks involving bulk samples and scRNA-seq data originating from different studies and sequencing methods. Moreover, CSsingle incorporates cell size coefficients, which enables the representation of the impact of cell size differences on deconvolution. In this work, we systematically evaluate the importance of incorporating cell size coefficients in cell type deconvolution. We demonstrate the effectiveness of CSsingle through comprehensive analyses conducted on a multitude of single-cell and bulk data sets encompassing gene expression profiles from pancreatic islet, peripheral blood mononuclear cells, whole blood, normal squamous-columnar junction, Barrett’s esophagus, and oesophageal carcinoma. Our results highlight the versatility and robustness of CSsingle as a potent tool for facilitating integrative analyses of bulk and scRNA-seq data across various biological and clinical studies.

## 2 Results

### 2.1 Existing deconvolution methods fail to consider differences in cell size

The deconvolution problem is typically cast in the form of a linear equation system: y=S×X, where X denotes the cell type composition from the bulk mixture y, and S denotes a signature matrix. Computational methods aim to deconvolve cell-type proportion X by using a signature matrix S. This matrix S consists of a set of marker genes discerned via differential expression analysis, which exhibit elevated expression in one cell population compared to others. Subsequently, the mean expression levels of these markers are calculated for each cell type, resulting in a N×K signature matrix. Consequently, the signature matrix S contains cell type-specific GEPs for the N selected marker genes across K cell types.

**Figure 1:**
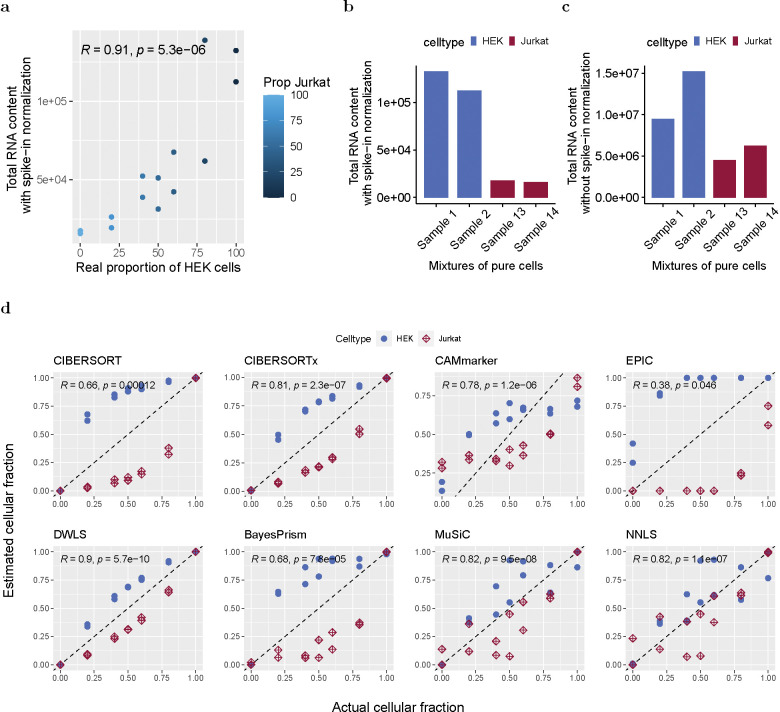
Cell type composition in mixtures of two cell types of markedly different cell sizes: HEK and Jurkat cells. **a** Scatter plot showing Pearson’s correlation between absolute RNA contents and real proportions of HEK cells for bulk mixtures. **b** Absolute RNA content estimated using ERCC spike-in controls for each cell type. **c** RNA content estimated by summing gene counts for each cell without ERCC spike-in normalization. **d** Performance of existing deconvolution methods on the mixtures of HEK (blue circles) and Jurkat (red squares) cells. The estimated cell-type proportions are plotted against the actual cell-type proportions. Reported ‘R’ corresponds to Pearson’s correlation and *p*-values indicate the significance of these correlations.

To build a signature matrix, existing deconvolution methods normally use single-cell GEPs measured by read/UMI counts, or TPM/CPM-normalized values [16–19]. We observed that these deconvolution methods by formulating y=S×X to estimate cellular fractions for bulk samples often introduce a systematic bias in the estimation when bulk samples consist of cell types with markedly different cell sizes which are commonly termed as the absolute cellular RNA contents. To illustrate this, we considered a real data set containing External RNA Controls Consortium (ERCC) spike-ins [22,23] introduced by Konstantin et al. [24], which was a compilation of 14 mixtures from two cell types (HEK and Jurkat cells) of markedly different cell sizes. Here we first estimated the absolute RNA content for each mixture by using ERCC spike-in controls to eliminate systematic technical differences in expression between mixtures, e.g. systematic variation introduced during the library preparations or sequencing of the samples. Each mixture in this data set, consisting of 10^6^ cells, is commonly assumed to contain similar total RNA contents by existing deconvolution methods employing TPM or CPM-normalized values. However, we observed that the total RNA content of each mixture after spike-in normalization significantly correlated with the proportions of HEK cells (Fig. 1a). In this data set, Samples 1, 2 and Samples 13, 14 are mixtures of pure HEK and Jurkat cells, respectively. Fig. 1b indicated that HEK cells had markedly larger cell sizes than Jurkat cells. Without spike-in normalization, the estimated cell size difference is less pronounced between the HEK and Jurkat cells than with spike-in normalization (Fig. 1b versus 1c), which is due to differences in technical effects introduced at the library preparation and sequencing levels.

For this real data set, we then used one mixture of pure HEK and two mixtures of pure Jurkat cells to generate cell type-specific GEPs to construct the signature matrix. One mixture of pure HEK cells, i.e. Sample 1 was excluded from the signature matrix construction due to technical bias (Fig. 1b). All 14 bulk mixtures were employed for benchmarking existing deconvolution methods against the ground truth. CIBERSORT, CIBERSORTx, CAMmarker [18], and EPIC [17] constructed signature matrices by normalizing the gene counts to CPM or TPM, while DWLS, BayesPrism [13], MuSiC and NNLS [10] used raw gene counts. As expected, all methods yielded estimated cellular fractions that systematically deviated from the actual cellular fractions (Fig. 1d). Specifically, for the deconvolution methods using a CPM or TPM-normalized signature matrix, cellular fractions of the HEK cell type, characterized by larger cell size, were consistently overestimated. Conversely, cellular fractions for the Jurkat cell type, with smaller cell size, were consistently underestimated (Fig. 1d, top panels). The potential source of this bias in cellular fraction estimations is often overlooked because of the commonly held, though rarely explicitly stated, assumption in deconvolution that the absolute amount of total mRNA is similar across different cell types. Both CPM and TPM-based methods intend to correct for library size using count depth scaling and assume that all cells are initially characterized by an equivalent quantity of mRNA molecules [21]. As a result, they produce an erroneous estimation of cellular fractions due to the marked difference in cell sizes between the two cell types. Alternatively, we also tested deconvolution methods using signature matrices of raw gene counts. As these methods do not correct for bias arising from “technical” library size, the cell sizes cannot be accurately estimated and the estimated cellular fractions did not align with the actual cellular fractions as well (Fig. 1d, bottom panels). These results highlighted the importance of incorporating cell size factors in computational deconvolution.

### 2.2 An iteratively reweighted least-squares approach to robustify deconvolution estimates

**Figure 2:**
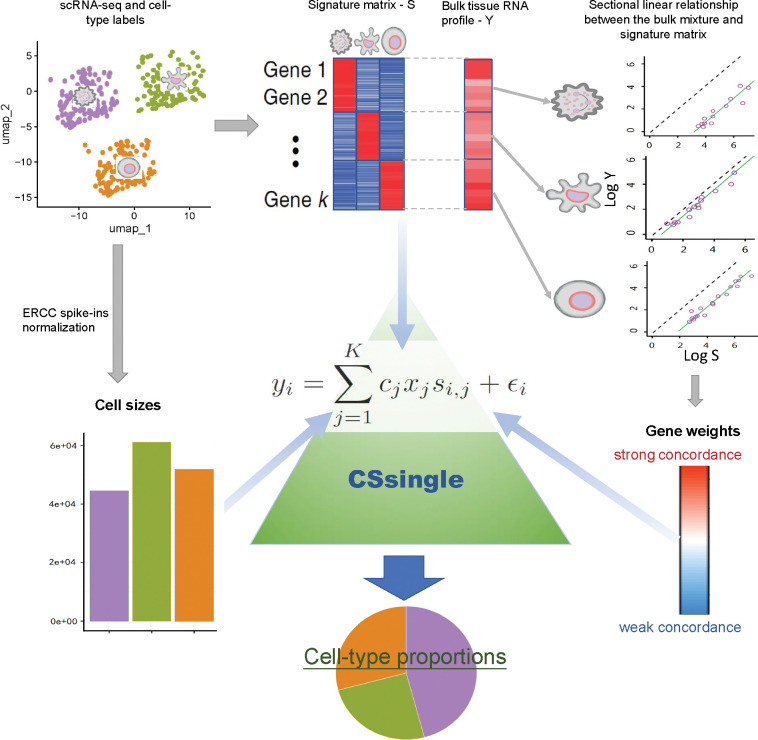
Schematic representation of the CSsingle decomposition method. CSsingle decomposes bulk transcriptomic data into a set of predefined cell types using the scRNA-seq or flow sorting reference. Within CSsingle, the cell sizes are estimated by using ERCC spike-in controls which allow the absolute RNA expression quantification. CSsingle is a robust deconvolution method based on the iteratively reweighted least squares approach. An important property of marker genes (i.e. there is a sectional linear relationship between the individual bulk mixture and the signature matrix) is employed to generate an efficient and robust set of initial estimates. The sectional linearity corresponds to the linear relationship between the individual bulk mixture and the cell-type-specific GEPs on a per-cell-type basis. CSsingle up-weights genes that exhibit stronger concordance and down-weights genes with weaker concordance between the individual bulk mixture and the signature matrix to accommodate technical and biological variations between the bulk and reference data. The detailed methods are described in the Methods section.

To recapitulate the effect of cell size differences on deconvolution, the gene expression of marker gene i in bulk mixture Y is modeled in CSsingle by $y_{i}=\sum_{j=1}^{K}\;c_{j}x_{j}s_{i,j}+\epsilon_{i}$ (Eq. 1). Here, $c_{j}$ denotes the cell size of cell type j, $x_{j}$ represents the fraction of cell type j in mixture Y, $s_{i,j}$ represents the column-normalized cell type-specific expression level of marker gene i within cell type j, and $\epsilon_{i}$ models measurement noise and other possible un-modeled factors. An overview of CSsingle is shown in Fig. 2. In this study, cell sizes were estimated by using a library of 96 external RNA spike-in controls developed by the External RNA Controls Consortium (ERCC) which enables researchers to utilize empirical evidence to eliminate systematic technical variations in expression between samples [22,23]. Our approach for inferring cell sizes relies on two assumptions: (i) the same amount of spike-in RNA is added to each cell; and (ii) technical effects should affect the spike-ins in the same way as they do the endogenous genes [25,26]. Under these assumptions, the ERCC spike-ins can act as technology-independent controls for cell size quantification.

Furthermore, when using scRNA-seq data to build a signature matrix for cell type deconvolution, it is important to account for variation between the bulk mixture and the signature matrix. Such variations can arise from technical and biological differences between the reference and bulk data, as previously indicated [19]. To appropriately use single-cell-derived cellular information to deconvolve bulk data, we designed a novel weighting scheme to solve cell type deconvolution as a minimization problem via $arg\;{min}_{X}\;\sum_{i=1}^{N}\;w_{i}(y_{i}-{\sum_{j=1}^{K}\;c_{j}x_{j}s_{i,j})}^{2}$ (Eq. 5). This scheme aims to appropriately adjust the contribution of each gene, taking into consideration potential influences from variations between the reference and bulk data.

CSsingle is a robust deconvolution method based on the concept of iteratively reweighted least squares (IRLS). To initialize an efficient and robust set of weights to solve the minimization problem, we relied on an important property of marker genes: there is a sectional linear relationship between the individual bulk mixture and the signature matrix. To illustrate this, we examined two fundamental assumptions underlying deconvolution methods: (i) expression profiles from each cell type are linearly additive (Eq. 1), and (ii) cell type-specific genes are exclusively or restrictively expressed in only one cell type within a bulk mixture. In an ideal scenario, genes exclusively expressed in one cell type scale exactly linearly with their expressions in a bulk mixture. We can thus write Eq. 1 as $log\;y_{i}=u_{j^{*}}+log\;s_{i,j^{*}}$ (Eq. 8), where $u_{j^{*}}=log(c_{j^{*}}x_{j^{*}})$, and marker gene i is exclusively expressed in cell type $j^{*}$. Based on Eq. 8, we thus identified this “sectional linear relationship” property of marker genes between an individual bulk mixture and the signature matrix. Specifically, for each cell type j, we employed a linear regression model to fit the bulk mixture Y and each cell-type-specific GEP within the signature matrix S using its marker genes in log scale. This model was represented as $log\;y_{i}=u_{j}+log\;s_{i,j}$ with the slope of exactly one, where $i\in {ℳ}^{j}$ and ${ℳ}^{j}$ is a finite set of marker genes for cell type j. Therefore, we determined the estimate $u_{j}$ for each cell type j using the respective fitted linear regression model. In Fig. 2, which provides a schematic illustration of CSsingle, we highlighted three cell types and fitted three linear regression models to represent the sectional linear relationship between the individual bulk mixture Y and the signature matrix S.

Once $u_{j}$ is estimated, CSsingle defines $\delta_{j}=\frac{exp\;u_{j}}{\sum_{j}\;exp\;u_{j}}$, representing the proportion of RNA content derived from cell type j in Y. In this formula, $exp\;u_{j}$ depends on $c_{j}x_{j}$ which represents the total RNA content derived from cell type j in the mixture Y. We then created an estimated bulk mixture based on $\delta_{j}$ as $Y^{*}={\left(t_{1},t_{2},\ldots ,t_{N}\right)}^{T}$ where $t_{i}=\sum_{j=1}^{K}\;\delta_{j}s_{i,j}$. Finally, we estimated the initial weight for each gene based on the difference between the real and estimated bulk mixtures, Y and $Y^{*}$. Specifically, CSsingle down-weights genes that have a big difference between Y and $Y^{*}$, a.k.a. the weak concordant genes between the individual bulk mixture Y and the signature mixture S. Down-weighting weak concordant genes is vital since this kind of genes are sensitive to the technical and biological differences between the bulk and reference data. Hence, CSsingle initializes an effective and resilient set of weights by assigning higher weights to marker genes exhibiting strong concordance and lower weights to genes with weak concordance. This approach offers a promising perspective for addressing technical and biological variations between bulk and single-cell reference data. The detailed method for further solving the minimization problem through IRLS is provided in the Methods section.

**Figure 3:**
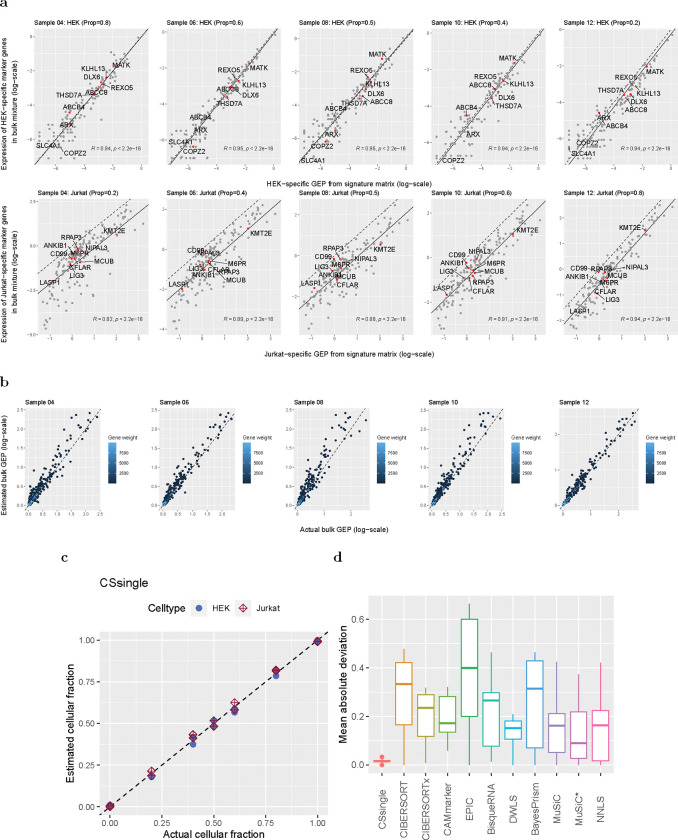
Application of CSsingle to deconvolution of bulk mixtures of HEK and Jurkat cells. **a** Linear regression demonstrating a slope of one between the individual bulk mixture and the cell type-specific GEPs for samples 04, 06, 08, 10, and 12. Top row: Linear regression between the mean expression levels of HEK-specific marker genes in the HEK-specific GEPs and their expressions in each bulk mixture. Bottom row: Linear regression between the mean expression levels of Jurkat-specific marker genes in the Jurkat-specific GEPs and their expressions in each bulk mixture. The dashed line in each plot represents the line of y=x. The signature matrix was constructed by selecting the top 150 marker genes for each cell type. Gene symbols of the top 10 most significant marker genes were plotted. **b** Scatter plots comparing estimated and actual bulk GEPs. The dashed line in each plot represents the line of y=x. **c** The plot illustrates the estimated cell-type proportions by CSsingle compared to the actual cell-type proportions. Reported ‘R’ corresponds to Pearson’s correlation and *p*-values indicate the significance of these correlations. **d** Boxplot depicting mean absolute deviation (MAD) between estimated and actual cell type proportions, with colors differentiating benchmark methods. The box encompasses quartiles of MAD, and whiskers span 1.5x the interquartile range.

To reveal and employ this important “sectional linear relationship” property of marker genes in real data by CSsingle, we first examined the linear relationship of marker genes in the aforementioned mixtures of HEK and Jurkat cells on a per-cell-type basis. We used mixtures of pure HEK or Jurkat cells to generate cell type-specific GEPs to construct the signature matrix. Within the signature matrix, cell type-specific marker genes were identified by using likelihood-ratio test. All bulk and single-cell data sets in this study are summarized in Table 1. In Fig. 3a, we specifically fitted a linear regression model with Eq. 8 (logy=u+logx) for each cell type using either HEK- or Jurkat-specific marker genes (solid lines). Importantly, for the five mixtures with different proportions of HEK and Jurkat cells, we observed a strong linear relationship between the HEK-specific GEPs from the signature matrix and the bulk mixture using HEK-specific marker genes (R>0.9; Fig. 3a, top panels, Samples 04, 06, 08, 10 and 12). We also observed a similar strong linear relationship between the Jurkat-specific GEPs from the signature matrix and the bulk mixture using Jurkat-specific marker genes (R>0.8; Fig. 3a, bottom panels). As a result, in these five bulk mixtures, we did reveal the sectional linear relationship between individual bulk mixtures and the signature matrix with two cell types.

To deconvolve the cell type proportions in all 14 different bulk mixtures, CSsingle takes advantage of the sectional linear relationship property to generate an efficient robust set of initial estimates by up-weighting marker genes with strong concordance and down-weighting genes with weak concordance (Fig. 3b) between the individual bulk mixture and the signature matrix. In addition, CSsingle estimated the cell sizes of HEK and Jurkat cells by using ERCC spike-ins (Fig. 1b) and incorporated the cell size coefficients in deconvolution. As shown in Fig. 3c–d, CSsingle accurately estimated the cellular fractions for deconvolving different mixtures of HEK and Jurkat cells, and its performance was significantly better than 10 other conventional methods.

### 2.3 CSsingle improves cross-platform deconvolution

For each cell type, we observed that their specific GEPs within the signature matrix were well-correlated with the healthy or T2D bulk mixture by applying linear regression to their marker genes in log-scale via Eq. 8 (*p* < 0.05; Fig. S1a,S2a). This analysis revealed that the bulk mixture was still sectional linear with the signature matrix though the two were generated from different sequencing methods and different disease states. However, considerable variations were observed for many genes. We therefore hypothesized that these observed variations were, to some extent, shaped by technical and biological differences between the bulk mixture and signature matrix derived from different sources. To take advantage of good concordant genes, we generated a set of initial coefficients by up-weighting marker genes with strong concordance and down-weighting genes with weak concordance between the bulk mixture and signature matrix (Fig. S1b,S2b). To assess the effectiveness of our initialization strategy, we also introduced a comparable step in CSsingle, where initial coefficients were estimated with constant gene weights. The results indicated a significant performance improvement when CSsingle up-weighted strong concordant genes and down-weighted weak concordant genes, as opposed to the initialization strategy using constant gene weights (Fig. S3).

**Figure 4:**
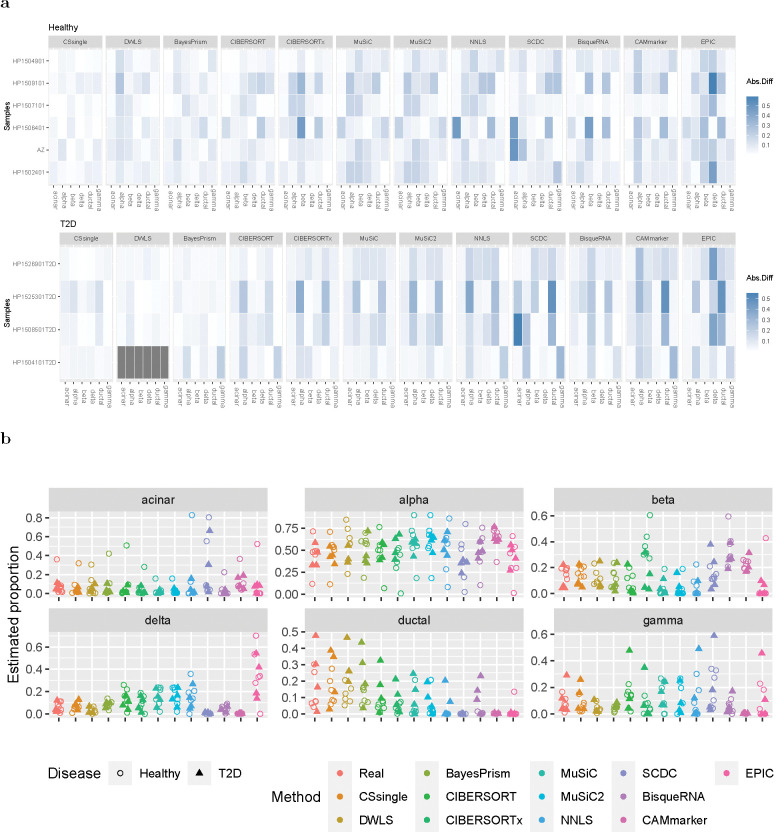
Decomposition benchmark in human pancreatic islet tissue. **a** Results of benchmarking deconvolution accuracy are depicted in heatmaps, illustrating the mean absolute deviation (mAD) between true and estimated cell type proportions in healthy (Top panel) and T2D (Bottom panel) samples. Darker colors represent higher mAD values. **b** Jitter plots displaying true and estimated cell type proportions. Each color represents a benchmarked method. Healthy subjects are denoted as dots while T2D subjects are denoted as triangles.

We further benchmarked the performance of CSsingle on the pancreatic islet data set by comparing the ground truth and cell type proportions estimated by 11 existing deconvolution methods. The pancreatic islet is a well-studied tissue with existing deconvolution methods [10,12,14,19]. A one-way ANOVA test indicated that there was no significant difference between the cellular RNA contents of six endocrine cell types in the scRNA-seq data set generated by Segerstolpe et al. [27] (p=0.47, Fig. S4). Therefore, the cell size coefficients of CSsingle were set to 1 for all cell types. The results showed that CSsingle achieved higher accuracy in terms of three metrics (root mean square deviation, mean absolute deviation, and Pearson’s correlation) over all other methods (Table S1). Although the signature matrix was only built from healthy samples, we found that CSsingle proved adept at accurately estimating the fractions of major and minor cell types for both healthy and T2D bulk mixtures (Fig. 4a,b). Specifically, all methods detected the alpha cell population as the predominant endocrine cell type, but MuSiC, MuSiC2, NNLS, and CAMmarker markedly overestimated the presence of alpha cells. Additionally, only CSsingle, DWLS, and BayesPrism could accurately estimate the proportion of ductal cells; other methods consistently underestimated its presence. We conducted a detailed examination of a minor cell type, delta cells. Among the methods assessed, CSsingle and BisqueRNA stood out as the only ones capable of accurately predicting the proportion of delta cells. Conversely, DWLS, SCDC, and CAMmarker consistently underestimated it, while BayesPrism, CIBERSORT, CIBERSORTx, MuSiC, MuSiC2, NNLS, and EPIC tended to overestimate its presence.

In practice, the single-cell reference data may encompass additional cell types absent from the bulk data. To assess the robustness of CSsingle and other methods in such a scenario, we conducted supplementary experiments. We preserved the synthetic bulk data and constructed a reference using scRNA-seq data from healthy donors across three studies: four cell types (alpha, beta, delta, and gamma) from Xin et al. [28], five cell types (alpha, beta, delta, acinar, and ductal) from Lawlor et al. [29], and nine cell populations (alpha, beta, delta, gamma, acinar, ductal, epsilon, endothelial and others) from Baron et al [30]. As shown in Table S2, only CSsingle accurately recovered the true cell type composition. Notably, CSsingle not only precisely estimated the proportions of the cell types (alpha, beta, delta, acinar, ductal, and gamma) present, but also those not present in the bulk data (epsilon, endothelial, and others).

### 2.4 CSsingle improves deconvolution across various single-cell sequencing methods

**Figure 5:**
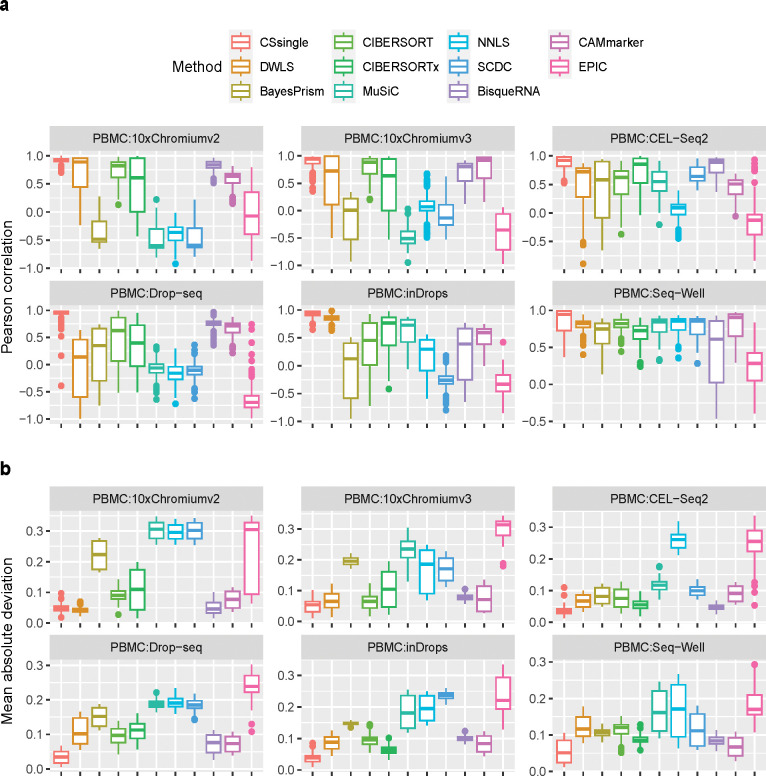
Decomposition benchmark in human PBMC. **a,b** Benchmarking of deconvolution accuracy in terms of Pearson’s correlation (**a**) and mAD (**b**). Data are depicted using boxplots, where the center line indicates the median, the box boundaries signify the upper and lower quartiles, and the whiskers span the full range of maximum and minimum values.

ScRNA-seq has become a pivotal tool for profiling cellular heterogeneity, with rapid development of new and improved laboratory protocols and associated transcript quantification tools. Thus, there is a need to assess performance of deconvolution methods for single-cell reference data generated from different laboratory protocols. To benchmark different deconvolution methods in this challenging but realistic scenario, we considered a benchmark data set introduced by Ding et al. [31], who were among the first to systematically examine how well scRNA-seq methods captured biological information. This data set profiled human peripheral blood mononuclear cells (PBMCs) samples employing two low-throughput plate-based methods, Smart-seq2 and CEL-Seq2, alongside five high-throughput methods: 10x Chromium v2, 10x Chromium v3, Drop-seq, Seq-Well and inDrops. We first created pseudo-bulk RNA-seq data by using single cells from PBMCs profiled by the SMART-Seq2 protocol. SMART-Seq2 prepares full-length libraries, which is essentially identical to the process used in bulk RNA-seq. We then built signature matrices using scRNA-seq data derived from six different scRNA-seq methods (10x Chromium v2, 10x Chromium v3, CEL-seq2, Drop-seq, inDrops, and Seq-Well), all of which incorporate UMIs. Since spike-in pools were not added to the background of this single-cell reference data, the cell size coefficients of CSsingle were set to 1 for all cell types. We compared the Pearson’s correlation and mean absolute deviation between actual and estimated cell type proportions across all 11 methods. As illustrated in Fig. 5, CSsingle achieved the best accuracy, while other methods performed substantially worse. We tested whether the mean cellular RNA contents of five immune cell types in the scRNA-seq data set used for pseudo-bulk construction were statistically different by a one-way ANOVA test. Fig. S5 shows that a statistically significant difference exists between cellular RNA contents of five immune cell types (*p* < 0.0001). Without introducing the cell size coefficients into CSsingle, we found that CSsingle achieved the best accuracy in recovering the proportions of B cells, Monocytes, CD4+ T cells and CD8+ T cells in terms of RMSD and mAD, while DWLS and CIBERSORTx performed best for NK cells (Table. S3).

### 2.5 CSsingle accurately estimates neutrophil-to-lymphocyte ratio from whole blood samples

Next, we evaluated the effectiveness of CSsingle in revealing cellular heterogeneity using clinical samples. We analyzed bulk transcriptomic data from a study involving influenza challenge in healthy adults aged 18–45, who were vaccinated with the A/Wisconsin/67/2005 (H3N2) strain. Genome-wide gene expression in their peripheral blood was assessed using Affymetrix microarrays at pre-challenge and on days 2–7 post-challenge [33]. Prior studies have shown that the neutrophil-to-lymphocyte ratio (NLR) was associated with disease severity and mortality for influenza patients [41,42], so being able to deconvolve neutrophils and lymphocytes in blood samples has significant implications in clinical applications. We conducted a concordance assessment between standard laboratory-derived neutrophil and lymphocyte proportions and estimates from deconvolution methods, using laboratory measurements sourced from a pre-existing study [43]. These measurements were recorded daily from day 1 to day 7, as well as a baseline measurement taken before inoculation. The PBMC data set from an RNA-seq study [32] served as the reference to deconvolve the whole blood samples. This reference data set contained 110 immune cells, which was divided into three large cell groups: neutrophils, lymphocytes, and myeloids. One-way ANOVA test revealed that there was no significant difference between the means of the three large cell groups being compared (*p* = 0.094, one-way ANOVA test; Fig. S6). Nevertheless, we observed the estimated cell size of myeloid cells are larger than those of other two cell groups.

**Figure 6:**
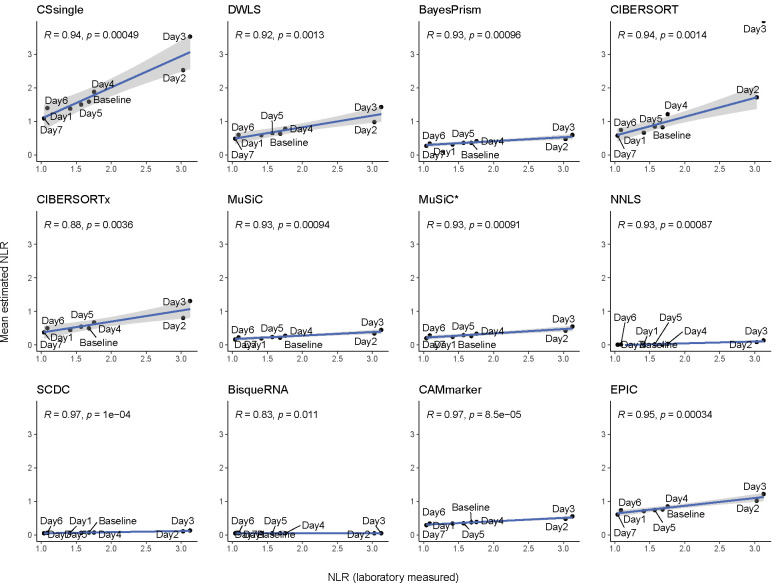
Correlation between laboratory-measured and estimated NLR proportions in the SI group of influenza H3N2. Data points are labeled by days post-inoculation, with the baseline denoting pre-inoculation and day 1 marking the day of inoculation. The ‘R’ value represents Pearson’s correlation coefficient, while *p*-values quantify the statistical significance of the observed correlations.

We compared the accuracy of all 12 methods in deconvolving neutrophils and lymphocytes using blood samples from symptomatic infected (SI) adults, individuals who exhibited noticeable signs or manifestations of the infection. The results showed CSsingle reached the best agreement between the estimated neutrophil/lymphocyte proportions and the gold standard (Fig. S7–S8). With the exception of the CAMmarker tool, which underestimated both neutrophil and lymphocyte proportions, the remaining methods consistently underestimated the neutrophil proportions and overestimated the lymphocyte proportions (Fig. S7–S8). This comparison demonstrated that the systematic bias could be corrected by introducing the cell size coefficients into the deconvolution analysis by CSsingle. We further benchmarked temporal changes in the NLR given its close association with the progression of influenza, as observed in existing clinical observations and studies [43]. The findings indicated that the temporal alterations in the NLR, as assessed by CSsingle, were congruent with those observed in laboratory white blood cell counts (R=0.94, p=0.00049; Fig. 6). While other existing methods also had a strong positive correlation between the estimated NLR and the laboratory measurements (R>0.8, p<0.05; Fig. 6), their NLRs were consistently underestimated. These results demonstrated that CSsingle correctly estimated the proportions of neutrophils and lymphocytes, as well as the NLR from whole blood samples, demonstrating its utility in clinical applications.

### 2.6 Application of CSsingle to Barrett’s esophagus

**Figure 7:**
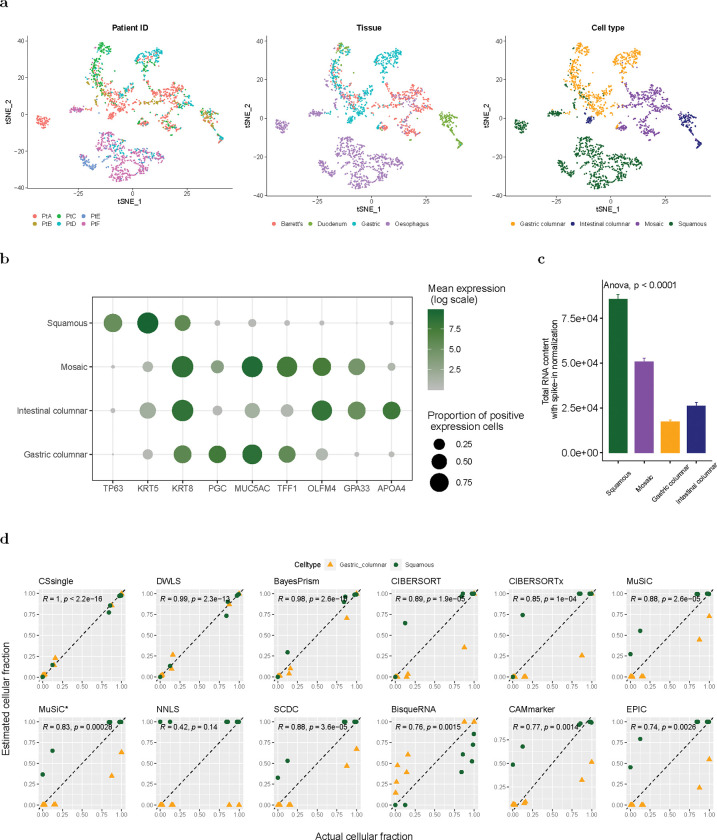
Decomposition benchmark in human normal squamous-columnar junction (N-SCJ) tissue. **a** t-SNE projection of scRNA-seq data from samples of BE, NE, NGC, and ND, and color coded by the patient ID (left panel), tissue (middle panel) and cell type (right panel). **b** Bubble chart of selected marker gene expression among different cell clusters. **c** Cell sizes estimated using ERCC spike-in controls for each cell type. Error bars represent standard deviation. One-way ANOVA test was used to test whether the estimated cell sizes of four epithelial cell populations are statistically different. **d** Performance of existing deconvolution methods on the simulated bulk data of N-SCJ. The plot compares deconvolution cell-type proportion estimates with the actual cell-type proportions, with colors indicating squamous (green) and gastric columnar (orange) cell types. The ‘R’ value represents Pearson’s correlation coefficient, while *p*-values quantify the statistical significance of the observed correlations.

We further extended our analysis to the epithelial compartment spanning the gastroesophageal junction. The native esophagus and gastric cardia are characterized as squamous and columnar, respectively. The squamous-columnar junction (SCJ), which delineates the boundary between esophageal and gastric epithelial cells, exhibits a mosaic of squamous and columnar cell types [34]. To examine the squamous and columnar compartments of SCJ, we built a signature matrix using a scRNA-seq data set derived from Barrett’s esophagus (BE), normal esophagus (NE), normal gastric cardia (NGC) and normal duodenum (ND) [35]. We then grouped the single cells into four epithelial cell populations: squamous, gastric, intestinal, and mosaic columnar (Fig. 7a). To achieve this categorization, the cellular components were first characterized by two cell types: squamous cells, identified by their marker genes TP63 and KRT5, and columnar cells, identified by the marker gene KRT8 [34] (Fig. 7b). In particular, the columnar cells were then further categorized into gastric, intestinal and mosaic columnar cells. The gastric and intestinal columnar cells were identified based on the restricted expression of markers such as PGC, MUC5AC, and TFF1 [44,45] for gastric columnar cells, and OLFM4, GPA33, and TFF3 [34] for intestinal columnar cells. Additionally, the mosaic columnar cells were defined by the co-expression of gastric and intestinal markers.

We estimated the cell sizes by using ERCC spike-in controls, and found that a statistically significant difference exists between the cell sizes of four epithelial cell populations (*p* < 0.0001, one-way ANOVA test; Fig. 7c). Specifically, the cell sizes of squamous cells are larger than columnar cells. To examine the squamous and columnar compartments of normal SCJ (N-SCJ), we created seven artificial bulk mixtures using a N-SCJ scRNA-seq data set [34]. This data set was generated from N-SCJ samples of seven healthy subjects. The signature matrix was built by selecting the squamous and gastric columnar cell populations shown in Fig. 7a. Our results demonstrated that the estimated cellular fractions by CSsingle correlated strongly with the actual cellular fractions, followed by DWLS and BayesPrism, while other methods performed substantially worse (Fig. 7d). Specifically, BisqueRNA overestimated cellular fractions of gastric columnar and underestimated cellular fractions of squamous, whereas other methods consistently overestimated cellular fractions of squamous and underestimated cellular fractions of gastric columnar.

**Figure 8:**
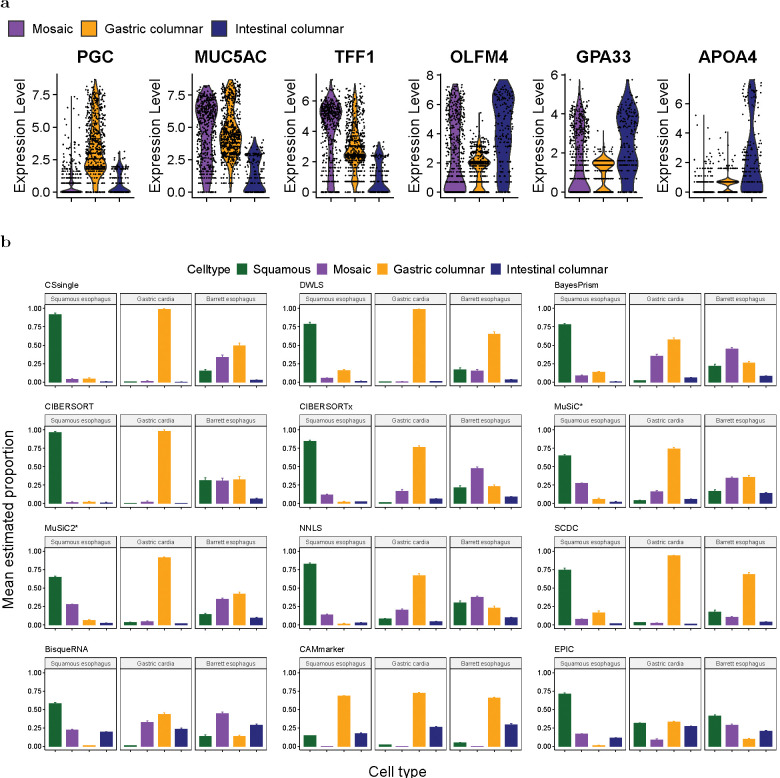
Decomposition benchmark in human Barrett’s esophagus tissue. **a** Violin plots of marker gene expression in mosaic, gastric, and intestinal columnar cells. **b** Comparison of decomposition estimates between CSsingle and other methods for 233 epithelium samples derived from NE, NGC and BE. Each color represents a cell cluster. Error bars represent standard deviation.

Barrett’s metaplasia is characterized by the transformation of squamous epithelium into columnar epithelium, serving as a precursor lesion for esophageal adenocarcinoma development [46]. In the case of BE, metaplasia occurs at the SCJ [47]. Prior investigations have reported that the individual columnar cells of BE showed a gastric and intestinal mosaic phenotype as defined by the co-expression of gastric and intestinal markers, while this mosaic phenotype was absent in the normal gastric or intestinal tissues [34]. Within our previously established signature matrix, we particularly identified a mosaic cell cluster that showed the co-expression of gastric markers (PGC, DMUC5AC, and TFF1) and intestinal markers (OLFM4, GPA33, and TFF3). This co-expression pattern was absent in the gastric or intestinal cell clusters (Fig. 8a).

To further estimate the cell composition in BE, we challenged CSsingle and 11 other deconvolution methods using 233 epithelium samples generated from NE, NGC, and BE tissues. Given that the NE and NGC tissues are squamous and columnar respectively, CSsingle, DWLS, CIBERSORT, CIBERSORTx, and SCDC identified the predominant cell type as squamous in NE and gastric columnar in NGC epithelium, while other methods either underestimated the squamous cells in NE or the gastric columnar cells in NGC (Fig. 8b). Furthermore, with the exception of SCDC, all other methods observed a substantial increase in the proportion of mosaic cell population in BE samples when compared to the proportions in NE and NGC (*p* < 0.05, one-sided Wilcoxon-test relative to NE and NGC, respectively; Fig. S9). The BE condition is characterized by the replacement of the oesophageal epithelium with a mosaic of gastric- and intestinal-like columnar cells [34,48]. As a result, these findings are consistent with clinical and histological observations, indicating gastric and intestinal phenotypic mosaicism in Barrett’s metaplasia.

### 2.7 Application of CSsingle to esophageal carcinoma

**Figure 9:**
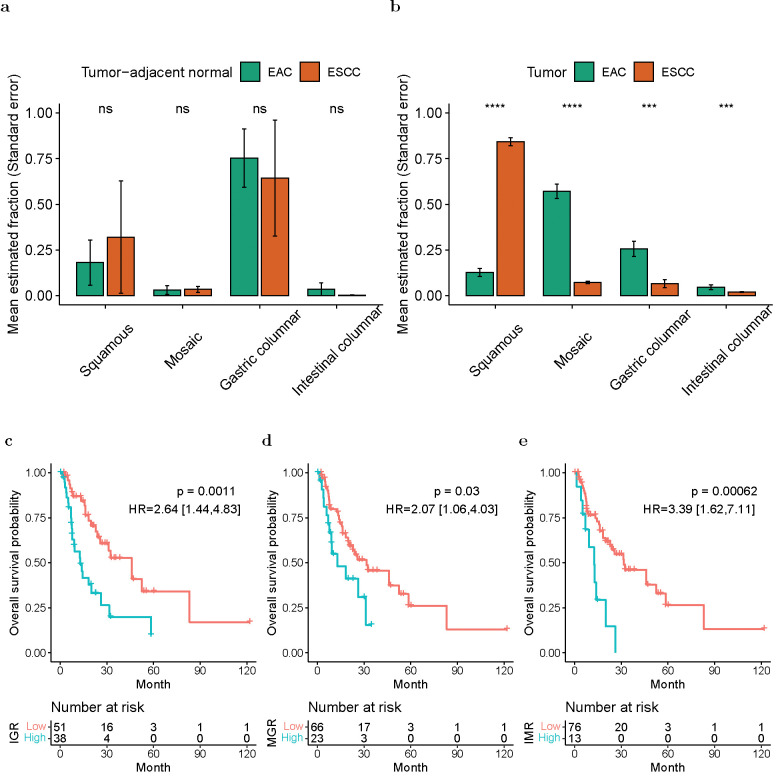
Application of CSsingle to deconvolution of oesophageal carcinoma biopsies. **a,b** The relative abundance of four epithelial cell populations for tumor-adjacent normal (**a**) and tumor (**b**) tissues in TCGA-ESAD cohort. Error bars represent standard deviation. Statistical significance between ESCC and EAC samples was assessed using a two-sided Wilcoxon test and indicated as follows: ${\;}^{ns}p\geq 0.05$, ^∗^*p* < 0.05,^∗∗^ *p* < 0.01,^∗∗∗^ *p* < 0.001, and ^∗∗∗∗^*p* < 0.0001. **c,d,e** Kaplan–Meier plot showing differences in overall survival between patients with esophageal adenocarcinoma having high and low estimated levels of IGR (**c**), MGR (**d**) or IMR (**e**). The statistical optimal cutoff was determined by analyzing quantiles ranging from the 10th to the 90th percentile in increments of 0.01, partitioning the cohort into two groups and selecting the cutoff that yielded the most significant p-value when assessing the risk disparity between these groups. Statistical significance was derived from the log-rank test. HR, hazard ratio. 95% HR confidence intervals are shown in square brackets.

Esophageal cancer is classified by histology as squamous cell carcinoma (ESCC) or adenocarcinoma (EAC), both of which share an anatomic site [46]. In this section, we applied CSsingle to dissect four major epithelial cell types - squamous, gastric, intestinal, and mosaic columnar - from the transcriptomes of 184 bulk oesophageal tumors and 11 adjacent normal tissues profiled by TCGA [40]. In the TCGA-ESAD data set, about 70% tumor samples were collected from the distal esophagus. Thus, for the deconvolution of tumor-adjacent normal tissues, we observed that the gastric columnar cells were consistently dominant in both the EAC and ESCC cohorts, followed by the squamous cells, while the presence of mosaic and intestinal columnar cells was rare (Fig. 9a). In addition, no significant differences in proportions of squamous, gastric, intestinal and mosaic columnar cells were observed for tumor-adjacent normal tissues between the EAC and ESCC cohorts (Fig. 9a). The progression of ESCC involves the sequential development of squamous hyperplasia, followed by low and high-grade squamous dysplasia, ultimately leading to invasive cancer [46]. In ESCC tumors we found that squamous cells were predominant (mean estimated proportions > 80%), with columnar cells comprising less than 20% of the total (Fig. 9b). In EAC, prior study revealed that the metaplastic epithelium which contains intestinal metaplasia, underwent a progression from low and high-grade dysplasia to invasive cancer [46]. We observed that the mosaic cell population was most abundant in EAC tumors (Fig. 9b), which shared the same characteristics as Barrett’s esophagus (BE). This result was also consistent with a previous study that revealed EAC occurred predominantly in the lower esophagus near the gastroesophageal junction and was associated with BE [40,49], indicating the mosaic cell population plays an important role in the progression to esophageal cancer.

Furthermore, we observed significantly higher gastric and intestinal columnar cells in EAC tumors than ESCC tumors (Fig. 9b). Previous studies revealed that EAC and intestinal-type gastric cancer shared many common molecular features [40,50]. We thus investigated whether the replacement of gastric columnar cells by cells of intestinal morphology is correlated with patient survival. To achieve this, we examined the association between IGR (intestinal-to-gastric columnar ratio), MGR (mosaic-to-gastric columnar ratio), IMR (intestinal-to-mosaic columnar ratio), and overall survival using a Cox proportional-hazards model. Our analysis revealed significant negative survival associations with IGR, MGR, and IMR (HR=2.64 [1.44,4.83], HR=2.07 [1.06,4.03] and HR=3.39[1.62,7.11], respectively; Fig. 9c,d,e). Taken together, these findings highlighted the ability of CSsingle to detect gastric-to-intestinal, gastric-to-mosaic, and mosaic-to-intestinal columnar replacement, which can inform clinical outcomes in esophageal adenocarcinoma.

### 2.8 Evaluation of signature matrix robustness

Utilizing a signature matrix not only improves computational efficiency but also significantly impacts deconvolution accuracy. The construction of a signature matrix is a process of feature selection. To achieve this, we first identified differentially expressed genes between each cell type and all other cell types based on the thresholds of an FDR adjusted p-value of less than 0.01 (calculated using Wilcoxon Rank Sum test unless otherwise specified) and a log2 mean fold change greater than 0.25. Next, for each cell type, we ranked the differentially expressed genes by their p-values and selected the top N genes to build the signature matrix. In this study, the value of N ranged from 50 to 300, incrementing by 50 at each step. We selected the optimal N for constructing a robust signature matrix by maximizing the Spearman’s correlation between the inferred and real bulk gene expression matrices (see Methods section). Traditionally, the stability of a signature matrix has been assessed using the 2-norm condition number, and the signature matrix with the minimum condition number was retained [11,16]. Here, we explored whether these two strategies achieved consistent results by integrating CSsingle with a signature matrix generated from each. We systematically evaluated the deconvolution results across 10 data sets encompassing HEK and Jurkat cell lines, pancreatic islet, PBMC and N-SCJ samples, which were studied in Fig. 3, 4, 5 and 7, and Table S2. The results showed that deconvolution results substantially improved and approximated ground truth cell proportions for the N-SCJ and PBMC:10xChrominumv3 data sets when selecting the signature matrix with an optimal N that maximized the Spearman’s correlation between the inferred and real bulk data. Across the remaining eight data sets, CSsingle exhibited comparable performance when using two different strategies for building the optimal signature matrix (Fig. S10). The results demonstrated that maximizing Spearman’s correlation between the inferred and real bulk data yielded a stable and robust signature matrix for deconvolution in CSsingle.

Furthermore, since computational efficiency and deconvolution accuracy also depend on the step size used in generating the final signature matrix, we conducted an additional experiment. We assessed the runtime and deconvolution results as the step size decreased from 50 to 1 on five data sets. Across five data sets, we found that setting the step size to 1 did not necessarily lead to improved accuracy. However, setting the step size to 50 achieved comparable performance while significantly enhancing computational efficiency and robustness of deconvolution (Table S4).

## 3 Discussion

Accurate cell-type deconvolution is critical for understanding cellular heterogeneity in clinical studies involving many samples. However, existing deconvolution methods fail with the decomposition of bulk mixtures consisting of cell types of markedly different cell sizes, which are referred to as the absolute cellular RNA contents. A fundamental assumption prevalent in high-throughput transcriptome analysis, such as cell type deconvolution, is that most genes exhibit consistent expression across various samples and cell types. Consequently, it is inferred that total cellular RNA levels remain constant. However, a substantial amount of evidence from various independent lines of research suggests that this assumption is frequently violated [51,52]. Thus, incorporating molecular spike-ins into cellular experiments can reveal the variability of genetic information across different cell types and enhance our understanding of the relationship between the quantity of cellular RNA molecules and other cellular characteristics, such as cell composition. Here, we introduce CSsingle, a computational method designed for the accurate decomposition of bulk transcriptomic data into a set of predefined cell types using the scRNA-seq or flow sorting reference. Through comprehensive benchmark evaluation and analysis of multiple real data sets, we underscore the importance of introducing cell size coefficients in computational deconvolution methods. CSsingle estimates cell size coefficients using the ERCC spike-in controls, enabling researchers to utilize empirical evidence to eliminate systematic technical variations in expression between samples. Absent the assumption of consistent total cellular RNA levels across different cell types, the use of cell size coefficients within the CSsingle method becomes imperative. Using the influenza challenge data set as an example, we have demonstrated that CSsingle can accurately predict the proportions of neutrophils and lymphocytes, as well as the NLR from whole blood samples while introducing the cell size coefficients.

CSsingle also facilitates robust estimation of cell composition for bulk mixtures and signature matrices derived from different sources. The iteratively reweighted least squares algorithm, integrated with robust initial estimates, efficiently addresses the technical and biological variations between individual bulk mixtures and the signature matrix. In both human pancreatic islet and PBMC datasets, we have demonstrated CSsingle’s ability to accurately predict cell composition for both bulk and single-cell reference data obtained from various laboratory protocols and disease states. Furthermore, our analysis extends to single-cell data from different RNA sequencing methods, affirming the broad applicability of CSsingle across different single-cell sequencing methods.

In this work, we also provide a comprehensive and biologically relevant validation of our predictions using more than 200 real diseased samples from Barrett’s esophagus and esophageal carcinoma. CSsingle estimations highlight the occurrence of phenotypic mosaicism in esophageal intestinal metaplasia. Moreover, our findings reveal that transitions such as gastric-to-intestinal, gastric-to-mosaic, and mosaic-to-intestinal columnar replacement can provide insights into clinical outcomes in esophageal adenocarcinoma.

CSsingle addresses several critical needs in the study of cellular heterogeneity: (i) it provides more accurate deconvolution of bulk RNA-seq and microarrays data compared to existing leading approaches, (ii) it introduces cell size coefficients in deconvolution, which properly corrects for bias arising from “technical” library size and “biological” cell size, and (iii) it effectively handles technical and biological variations between individual bulk mixtures and the signature matrix. However, in practical applications, the precision of CSsingle may be compromised when certain cell types are absent from the reference matrix, a limitation shared by all deconvolution techniques. We stress that our study specifically evaluated the efficacy of utilizing ERCC spike-in controls for cell size quantification. We did not, however, examine the robustness of spike-ins when used for “absolute” quantification, the process intended to specify the exact quantity of each transcript’s molecules within individual cells.

In conclusion, CSsingle emerges as an accurate and robust deconvolution approach that holds promise in elucidating mechanisms underlying cellular heterogeneity, which often obscured within existing large cohorts of bulk transcriptomic data.

## 4 Methods

### 4.1 Construction of the signature matrices

The majority of current cell type deconvolution techniques, which depend on a signature matrix composed of cell type-specific GEPs, operate under the premise that cells can be categorized into a predetermined set of types. They also assume that the prevalent cell types within the bulk tissue are adequately reflected in the scRNA-seq data. To build a signature matrix from scRNA-seq data, we started with a read or UMI count matrix. We first summed up gene counts from all cells assigned to the same cell type. The summation was followed by normalization based on total count and multiplication by a scale factor of 10^4^. This process produced a matrix of genes × cell types (denoted as $\tilde{S}$), from which a sub-matrix (denoted as S) was derived by selecting a set of differentially expressed genes. The identification of differentially expressed genes was accomplished using the FindAllMarkers function with the default parameters of the Seurat R package (version 5.0.1). Specifically, we initially isolated differentially expressed genes using a log-scale threshold of ≥ 0.25-fold over-expression in a given cell population relative to all others. Subsequently, non-significant genes with a p-value larger than 0.01 (Wilcoxon Rank Sum test unless otherwise specified) were filtered out. Next, we ranked the differentially expressed genes in ascending order by their p-values and selected the top *N* marker genes for each cell type as the most significantly differential expressed marker genes. Additionally, we excluded marker genes shared between two or more cell types. We generated multiple signature matrices by varying N from 50 to 300 with step 50 by default.

### 4.2 CSsingle model

CSsingle is an iteratively reweighted linear-regression model that decomposes bulk gene expression data into a set of predefined reference cell types to estimate cell abundances. In order to accurately estimate the cell type fraction, we designed a novel weighting scheme to properly adjust the contribution of each marker gene. We denote $\tilde{Y}$ as a bulk mixture. Both $\tilde{S}$ and $\tilde{Y}$ consist of column-normalized expression values, so that every cell type and bulk mixture have the same total count of 10^4^ across all genes shared between the scRNA-seq and bulk data. Additionally, we denote the truncated version of $\tilde{S}$ and $\tilde{Y}$, containing only N significant differential expressed marker genes, as S and Y, respectively. Let $S=\left[\begin{array}{ccc} s_{11} & \cdots & s_{1K}\\ \vdots & \ddots & \vdots \\ s_{N1} & \cdots & s_{NK} \end{array}\right]$ and $Y={\left(y_{1},y_{2},\ldots ,y_{N}\right)}^{T}$. The deconvolution model for an observed bulk mixture within CSsingle is defined as follows:

(1) $$y_{i}=\sum_{j=1}^{K}c_{j}x_{j}s_{i,j}+\epsilon_{i}$$

(2) $$\epsilon_{i}∼N\left(0,\sigma^{2}\right)$$

(3) $$\forall j\;:x_{j}\geq 0$$

(4) $$\sum_{j=1}^{K}x_{j}=1$$

where S is an N×K signature matrix that contains GEPs for the N marker genes across K cell types, Y is an N×1 vector representing a single bulk gene expression profile for the same N marker genes, $X={\left(x_{1},x_{2},\ldots ,x_{K}\right)}^{T}$ is a K×1 vector containing the cell type composition from the bulk gene expression data Y, $c_{j}$ denotes the cell size of cell type j, and ϵ models measurement noise and other possible un-modeled factors. The constraints in (3) and (4) require the cell type fractions to be positive and to sum up to one.

Within CSsingle, we introduce the cell size factor $c_{j}$ to consider differences in cell size and their effect on the deconvolution problem. Cell sizes are estimated by using ERCC spike-in controls which allow absolute RNA expression quantification. Specifically, we normalize gene counts by the upper-quartile (UQ; 75th percentile by default) of the ERCC spike-in counts to adjust for varying sequencing depths and other potential technical effects. The absolute cellular RNA content is calculated by summing the normalized gene counts in each cell. We define the cell size $c_{j}$ as the mean value of absolute cellular RNA contents in each cell of cell type j.

To minimize the technical and biological variation between the bulk mixture and reference matrix, CSsingle introduces $w_{i}$ by up-weighting marker genes with strong concordance and down-weighting genes with weak concordance between the individual bulk mixture and signature matrix. To estimate X and $\overset{ˆ}{X}$, CSsingle minimizes the weighted squared error as follows:

(5) $$\hat{X}=\arg\underset{X}{\min}\sum_{i=1}^{N}w_{i}{\left(y_{i}-\sum_{j=1}^{K}c_{j}x_{j}s_{i,j}\right)}^{2}$$

(6) $$\text{s.t}\;\forall j\;:x_{j}\geq 0$$

(7) $$\sum_{j=1}^{K}x_{j}=1$$

Here, the weights $w_{i}$ are interdependent with the residuals and the estimated coefficients, forming an iterative dependency loop. We thus employed an iterative approach (called iteratively reweighted least-squares, IRIS) to solve this weighted-least-squares problem.

To achieve an efficient and robust estimation, it is vital to give careful consideration to the initial estimates. CSsingle takes advantage of the sectional linear relationship of the marker genes to generate an efficient set of initial estimates. We define ${ℳ}^{j}$ as a finite set of marker genes for cell type j, where j=1,2,...,K. Let $Nj\;=\;|{ℳ}^{j}\;|$ represent the total number of marker genes for cell type j, and consequently, $N=\sum_{j}\;N_{j}$. For each cell type j, CSsingle employs a linear regression model to fit the bulk mixture Y and each cell type-specific GEPs within signature matrix S with its marker genes in log-scale using Eq. 8. The goal is to find the best-fitting curve with a regression slope of one to a data set comprising Nj observations of cell type-specific gene expression values ${\left(log\;s_{i,j}\right)}_{i\in {ℳ}^{j}}^{T}$, together with corresponding observations of the bulk gene expression values of ${\left(log\;y_{i}\right)}_{i\in {ℳ}^{j}}^{T}$ by minimizing the sum of the squares of the offsets of the points from the curve.

(8) $$\log y_{i}=u_{j}+\log s_{i,j}\;\left(i\in {ℳ}^{j}\right),$$

where $u_{j}=log\left(c_{j}x_{j}\right)$ is the disturbance term for cell type j estimated from the above linear regression with a slope of one. We then define $\delta_{j}=\frac{exp\;u_{j}}{\sum_{j}\;exp\;u_{j}}$, representing the proportion of RNA content derived from cell type j in Y, where $expu_{j}=c_{j}x_{j}$ represents the total RNA content derived from cell type j in Y. Next, we use $\delta_{j}$ to generate an estimated bulk mixture as $Y^{*}={\left(t_{1},t_{2},\ldots ,t_{N}\right)}^{T}$, where $t_{i}=\sum_{j=1}^{K}\;\delta_{j}s_{i,j}$. Finally, the initial weight for gene i is defined based on the difference between Y and $Y^{*}$:

(9) $$w_{i}^{\left(0\right)}=\frac{1}{{\left(y_{i}\;-\;t_{i}\right)}^{2}},\;\text{for}\;i=1,\;2,\ldots ,N$$

Let:

(10) $$X^{\left(0\right)}=\arg\underset{X}{\min}\sum_{i=1}^{N}w_{i}^{\left(0\right)}{\left(y_{i}-\sum_{j=1}^{K}c_{j}x_{j}s_{i,j}\right)}^{2},$$

(11) $$X^{\left(1\right)}=\arg\underset{X}{\min}\sum_{i=1}^{N}w_{i}^{\left(1\right)}{\left(y_{i}-\sum_{j=1}^{K}c_{j}x_{j}s_{i,j}\right)}^{2},\;\mathrm{where}\;w_{i}^{\left(1\right)}=\frac{1}{{\left(y_{i}\;-\;\sum_{j=1}^{K}c_{j}x_{j}^{\left(0\right)}s_{i,j}\right)}^{2}},$$

(12) $$X^{\left(t\right)}=\arg\underset{X}{\min}\sum_{i=1}^{N}w_{i}^{\left(t\right)}{\left(y_{i}-\sum_{j=1}^{K}c_{j}x_{j}s_{i,j}\right)}^{2},\;\mathrm{where}\;w_{i}^{\left(t\right)}=\frac{1}{{\left(y_{i}\;-\;\sum_{j=1}^{K}c_{j}x_{j}^{\left(t-1\right)}s_{i,j}\right)}^{2}}.$$

The estimated coefficients converge when $∥X^{\left(t\right)}-X^{\left(t-1\right)}∥\leq 0.01$, and the optimal $X^{\left(t\right)}$ is the final cell type composition estimated from the bulk data.

### 4.3 Additional adjustments to improve performance for microarray

Given the major differences between RNA sequencing and microarray techniques, deconvolution might prove ineffective in scenarios where excessive technical variation exists. For microarray, background hybridization and probe saturation can impede the detection of transcripts at both low levels and high levels. In contrast, RNA sequencing enables the detection of a broader range of transcripts, including those with low abundance and high abundance [53,54]. We, therefore developed a strategy for handling bulk mixtures derived from microarrays, tailored for signature matrix generated from RNA sequencing, or vice versa. We introduced an upper bound q, which limits the maximum value that any weight can take on. The adjusted weights are defined as:

(13) $${\overset{ˆ}{w}}_{i}=\left\{\begin{array}{l} w_{i},\;\text{if}\;w_{i}<q;\\ q,\;\text{otherwise.} \end{array}\right.$$

The upper bound q is selected as follows. The possible values for q are defined as the $\tau^{th}$ quantile of the gene weights ${\left(w_{1},w_{2},\cdots ,w_{N}\right)}^{T}$, where τ is selected from 0.01 to 1 with a step of 0.01. For each possible value of q, we obtain an estimate for X, denoted as $\overset{ˆ}{X}={\left({\overset{ˆ}{x}}_{1},{\overset{ˆ}{x}}_{2},\cdots ,{\overset{ˆ}{x}}_{K}\right)}^{T}$, by minimizing the weighted squared error with respect to the adjusted weights ${\left({\overset{ˆ}{w}}_{1},{\overset{ˆ}{w}}_{2},\cdots ,{\overset{ˆ}{w}}_{N}\right)}^{T}$:

(14) $$\hat{X}=\arg\;\underset{X}{\min}\sum_{i=1}^{N}{\hat{w}}_{i}{\left(y_{i}-\sum_{j=1}^{K}c_{j}x_{j}s_{i,j}\right)}^{2}.$$

Subsequently, the inferred bulk gene expression profile $\overset{ˆ}{Y}={\left({\overset{ˆ}{y}}_{1},{\overset{ˆ}{y}}_{2},\cdots ,{\overset{ˆ}{y}}_{N}\right)}^{T}$ is defined as follows:

(15) $${\hat{y}}_{i}=\sum_{j=1}^{K}c_{j}{\hat{x}}_{j}s_{i,j}.$$

CSsingle calculates the Spearman’s correlation between the inferred and real bulk gene expression profiles, $\overset{ˆ}{Y}$ and Y. The value of q corresponding to the maximum correlation coefficient is then selected.

### 4.4 Selecting the optimal signature matrix

In Methods section 4.1, we describe the construction of multiple signature matrices by CSsingle. To determine the optimal signature matrix, CSsingle is integrated with each signature matrix to estimate the cell type proportions. The optimal signature matrix $S^{*}$ is selected as the candidate matrix whose inferred bulk gene expression matrix has the highest Spearman’s correlation coefficient with the real bulk gene expression matrix.

### 4.5 Construction of artificial bulk data sets

The aggregated counts for the synthetic bulk dataset are derived from a scRNA-seq dataset, with the bulk counts computed as the sum of gene counts across all cells within the same sample. Specifically, for the scRNA-seq data set spanning fewer than five samples, we generate t bootstrap replicates, each of which matches the size of the original scRNA-seq data set by randomly sampling cells with replacement. Next, counts for the artificial bulk data set are generated from each bootstrap replicate by summing up gene counts from all cells within the same sample. For the artificial bulk data set in Fig. 5, we used t=100. The actual cellular proportion of cell type j in the sample s is calculated by

(16) $${\overset{ˆ}{x}}_{j}^{s}=\frac{n_{j}^{s}}{\sum_{j}\;\;n_{j}^{s}}.$$

where $n_{j}^{s}$ is the number of cells for cell type j in sample s.

### 4.6 Systematic evaluation of CSsingle and comparison against baseline methods

In this study, we benchmarked the performance of CSsingle against 11 existing methods: DWLS, BayesPrism, CIBERSORT, CIBERSORTx, MuSiC, MuSiC2, NNLS, SCDC, BisqueRNA, CAMmarker, and EPIC. Further details regarding their implementation and specific parameters can be found in the respective original publications and GitHub repositories.

- **DWLS**. We downloaded DWLS from https://bitbucket.org/yuanlab/dwls/src/default/. We used raw gene count matrices as input for both single-cell reference and bulk data. The signature matrix was constructed using the hurdle model in the MAST R package, setting all parameters to their default values. In the event of negative values, the estimated proportions were adjusted to zero. When the function solve.QP fails to find a solution due to inconsistent constraints, DWLS outputs errors.
- **BayesPrism**. The R package BayesPrism was downloaded from https://github.com/Danko-Lab/BayesPrism.git. We used raw count matrices as input for single-cell reference and bulk data, setting all parameters to their default values.
- **CIBERSORT**. CIBERSORT was run online (https://cibersortx.stanford.edu/runcibersortx.php). We used raw count matrices as input for both single-cell reference and bulk data, setting all parameters to their default values. Additionally, no batch correction was applied.
- **CIBERSORTx**. CIBERSORTx was executed online (https://cibersortx.stanford.edu/runcibersortx.php). We used raw count matrices as input for both single-cell reference and bulk data, setting all parameters to their default values. Additionally, batch correction was applied to reduce cross-platform variance.
- **MuSiC**. The R package MuSiC was downloaded from https://github.com/xuranw/MuSiC. The single-cell reference and bulk data were used as input. MuSiC was employed with parameter ‘cell size’ set as NULL (default value, estimating cell size from data), while MuSiC* was employed with ‘cell size’ estimated using ERCC spike-in controls. All other parameters were left at their default values.
- **MuSiC2**. The MuSiC2 functions are available within the R package MuSiC. MuSiC2 was designed for the deconvolution of multi-condition bulk data, we thus ran it only for multi-condition bulk data. We followed the tutorials of MuSiC2 on GitHub, and the single-cell reference and bulk data were used as input. MuSiC2 was employed with parameter ‘cell size’ set as NULL (default value, estimating cell size from data), while MuSiC2* was employed with ‘cell size’ estimated using ERCC spike-in controls. All other parameters were left at their default values.
- **NNLS**. The NNLS method is implemented using the R package MuSiC. We used the single-cell reference and bulk data as input. All parameters were set to their default values.
- **SCDC**. The R package SCDC was downloaded from http://meichendong.github.io/SCDC. We used both single-cell reference and bulk data as input. During the quality control procedure, we set the parameter ‘qcthreshold = 0.7’, while leaving all other parameters at their default values.
- **BisqueRNA**. The R package BisqueRNA was downloaded from https://github.com/cozygene/bisque. We used both single-cell reference and bulk data as input. All parameters were set to their default values.
- **CAMmarker**. The Bioconductor R package debCAM was downloaded from https://bioconductor.org/packages/release/bioc/html/debCAM.html. We used the CPM or TPM normalized expression values of bulk data as input. The parameter ‘MGlist’ defines a list of vectors, each containing known markers for one cell type. These markers are chosen from the candidate signature matrix with the lowest condition number.
- **EPIC**. The R package EPIC was downloaded from https://github.com/Gfeller-Lab/EPIC. The CPM or TPM normalized expression values of bulk data were used as input. The parameter ‘sigGenes’ defines a character vector consisting of gene names chosen to serve as a signature for the deconvolution process. These genes are selected from the candidate signature matrix with the lowest condition number.

### 4.7 Assessment of deconvolution performance

We assessed the performance of various deconvolution methods using Pearson’s correlation coefficient (R), root mean squared deviation (RMSD), and mean absolute deviance (mAD) as evaluation metrics. These metrics are calculated using the following equations:

(17) $$R=Cor(X,\overset{ˆ}{X}),\;RMSD=\sqrt{Avg(X-\overset{ˆ}{X}{)}^{2}},mAD=Avg(|X-\overset{ˆ}{X}|).$$

where X and $\overset{ˆ}{X}$ are actual and estimated cell type proportions, respectively.

### 4.8 Statistical analysis

All statistical analyses were conducted using R (version 4.3.1; available at https://cran.r-project.org/). Specific statistical tests are detailed in the figures and their respective captions when applicable. A *p-*value threshold of 0.05 was used to determine statistical significance for all tests, unless otherwise specified. Statistical significance between ESCC and EAC samples was assessed using a two-sided Wilcoxon test and reported as follows: ${\;}^{ns}p\geq 0.05,$ *p < 0.05, **p < 0.01, ***p < 0.001, and ^∗∗∗∗^*p* < 0.0001. The cumulative survival time was estimated via the Kaplan–Meier method, with the log-rank test from the R survminer package employed to assess survival curve disparities.

## Supplementary Material

Supplement 1

## Figures and Tables

**Table 1:** Details of the data sets used in the study.

Tissue type	Data source	Platform	Protocol	Data type	# Cell types	# Samples	# Cells	# Genes	ERCC spike-ins availability

Cell line	GSE129240 [24]	Illumina HiSeq 2500	NA	RNA-seq	2	13	13	35022	Yes
Pancreatic islet	E-MTAB-5061 [27]	Illumina HiSeq 2000	Smart-seq2	scRNA-seq	6	10(6H+4T2D)	2038	25526	No
	GSE81608 [28]	Illumina HiSeq 2500	Fluidigm C1	scRNA-seq	4	18(12H+6T2D)	1492	39849	No
	GSE86473 [29]	Illumina NextSeq 500	Fluidigm C1	scRNA-seq	5	8(5H+3T2D)	580	26544	No
	GSE84133 [30]	Illumina Hiseq 2500	inDrop	scRNA-seq	14	4(3H+1T2D)	8569	20125	No
PBMC	GSE132044 [31]	Illumina HiSeq 2500	Smart-seq2 10x Chromium v2 10x Chromium v3 CEL-seq2 Drop-seq inDrops Seq-Well	scRNA-seq	5	14	29401	33694	No
	GSE107011 [32]	Illumina HiSeq 2000	Smart-seq2	RNA-seq	28	110	127	51518	Yes
Whole blood	GSE73072 [33] (H3N2,SI)	GeneChip Human Genome U133A 2.0	Affymetrix	Microarray	NA	371	NA	11864	No
N-SCJ	EGAS00001000723 [34] (cellxgene,N-SCJ)	Illumina HiSeq 2000	10x Chromium	scRNA-seq	2	7	10426	23327	No
NE,NGC,ND,BE	EGAS00001003144 [35] (Patients A-F)	Illumina HiSeq 4000	Smart-seq2	scRNA-seq	4	17	2340	33151	Yes
NE,NGC,BE	GSE34619 [36]	Human Gene 1.0 ST	Affymetrix	Microarray	NA	28(8NE+10NGC+10BE)	NA	18778	No
	GSE39491 [37]	Human Genome U133A 2.0	Affymetrix	Microarray	NA	120(40NE+40NGC+40BE)	NA	12645	No
NE,BE	GSE36223 [38]	Human Genome U133A 2.0	Affymetrix	Microarray	NA	46(23NE+23BE)	NA	13041	No
	GSE26886 [39]	Human Genome U133 Plus 2.0	Affymetrix	Microarray	NA	39(19NE+20BE)	NA	21367	No
Oesophageal carcinoma	TCGA-ESCC [40]	Illumina HiSeq 2000	NA	RNA-seq	NA	98(95T+3N)	NA	20501	No
	TCGA-EAC [40]	Illumina HiSeq 2000	NA	RNA-seq	NA	97(89T+8N)	NA	20501	No

## Data Availability

All data analyzed in this study are publicly available through online sources. Accession numbers and reference links to all data sources are presented in Table 1.
