# Supplementary material for: Leveraging cross-source heterogeneity to improve the performance of bulk gene expression deconvolution
Source: bioRxiv. 2024 Apr 9:2024.04.07.588458. Preprint. [Version 1] doi: 10.1101/2024.04.07.588458 (PMC11030304; doi:10.1101/2024.04.07.588458)
Supplement: Supplementary file 1 [file media-1.pdf]

Supplementary Information

Wenjun Shen[1,*], Cheng Liu[2], Yunfei Hu[3], Yuanfang Lei[1], Hau-San Wong[4], Si Wu[5,*], and Xin Maizie Zhou[3,6,*]

Department of Bioinformatics, Shantou University Medical College, Shantou, Guangdong 515041, China
Department of Computer Science, Shantou University, Shantou, Guangdong 515041, China
Department of Computer Science, Vanderbilt University, Nashville, TN 37235, USA
Department of Computer Sciences, City University of Hong Kong, Kowloon, Hong Kong
Department of Computer Science, South China University of Technology, Guangzhou, Guangdong 510006, China
Department of Biomedical Engineering, Vanderbilt University, Nashville, TN 37235, USA
wjshen@stu.edu.cn, cswusi@scut.edu.cn, maizie.zhou@vanderbilt.edu

a

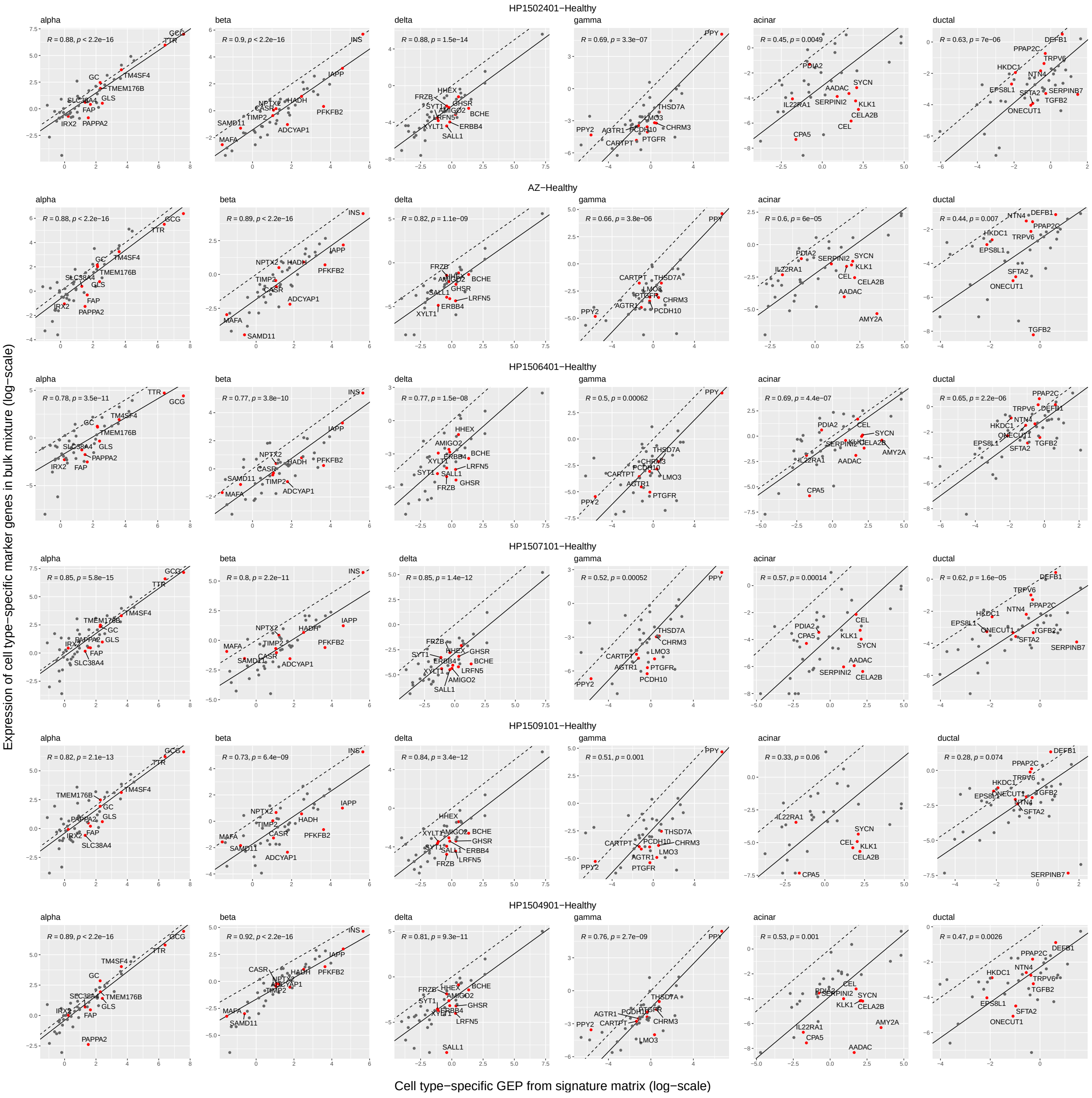

b

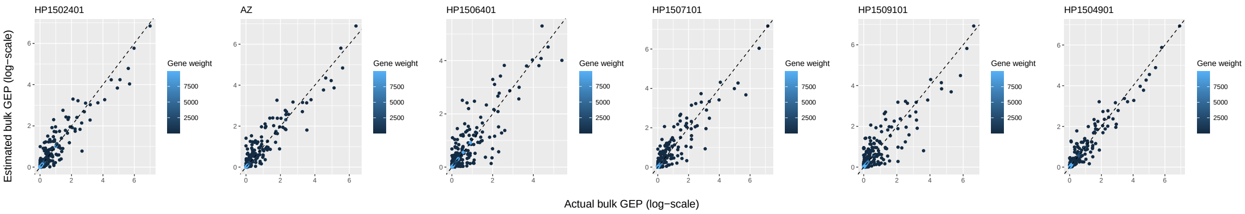

Figure S1: Linear relationship for healthy samples in the pancreatic islet data set. **a** Linear regression demonstrating a slope of one between individual bulk mixtures and cell type-specific GEPs for healthy pancreatic islet samples. The dashed line in each plot represents the line of $y = x$. The signature matrix was constructed by selecting the top 50 marker genes for each cell type. Gene symbols of the top 10 most significant marker genes were plotted. **b** Scatter plots comparing estimated and actual bulk GEPs. The dashed line in each plot represents the line of $y = x$.

a

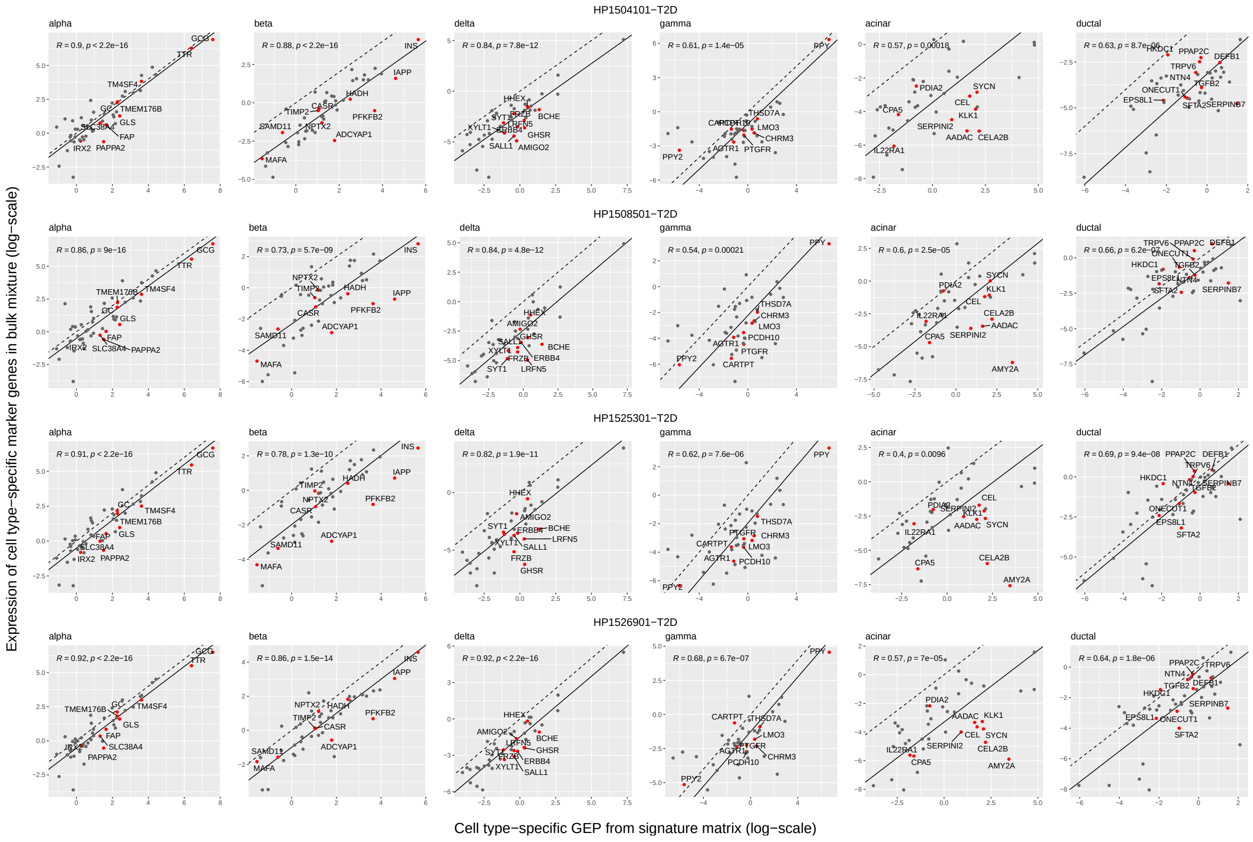

b

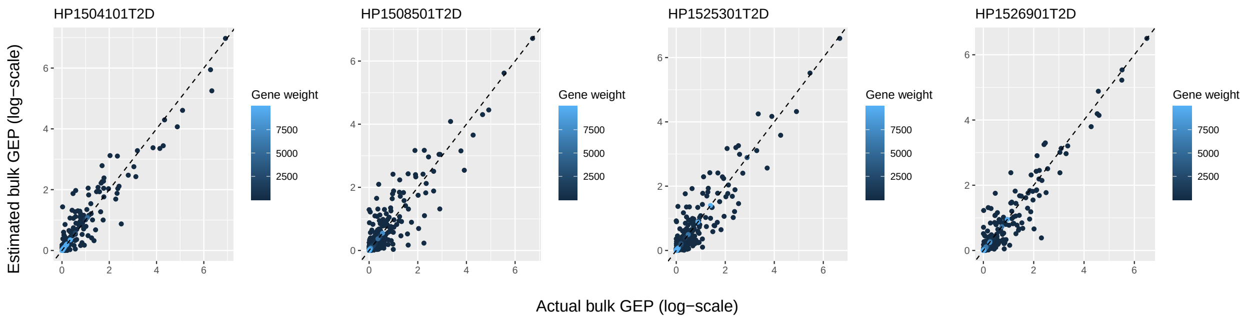

Figure S2: Linear relationship for T2D samples in the pancreatic islet data set. **a** Linear regression demonstrating a slope of one between individual bulk mixtures and cell type-specific GEPs for T2D pancreatic islet samples. The dashed line in each plot represents the line of $y = x$. The signature matrix was constructed by selecting the top 50 marker genes for each cell type. Gene symbols of the top 10 most significant marker genes were plotted. **b** Scatter plots comparing estimated and actual bulk GEPs. The dashed line in each plot represents the line of $y = x$.

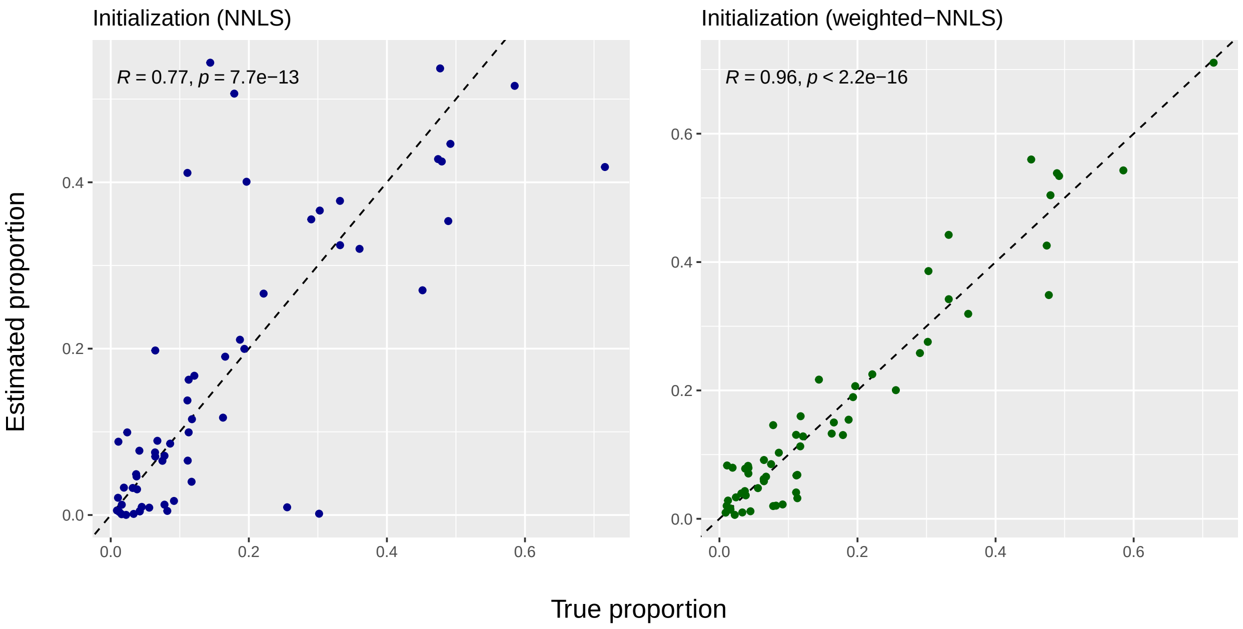

Figure S3: **Comparison of the performance of CSsingle initialized with two different weighting strategies for pancreatic islet samples.** Left panel: constant gene weights (NNLS) and Right panel: more weights on strong concordant genes and less weights on weak concordant genes (weighted-NNLS). Reported 'R' corresponds to Pearson's correlation and p-values indicate the significance of these correlations.

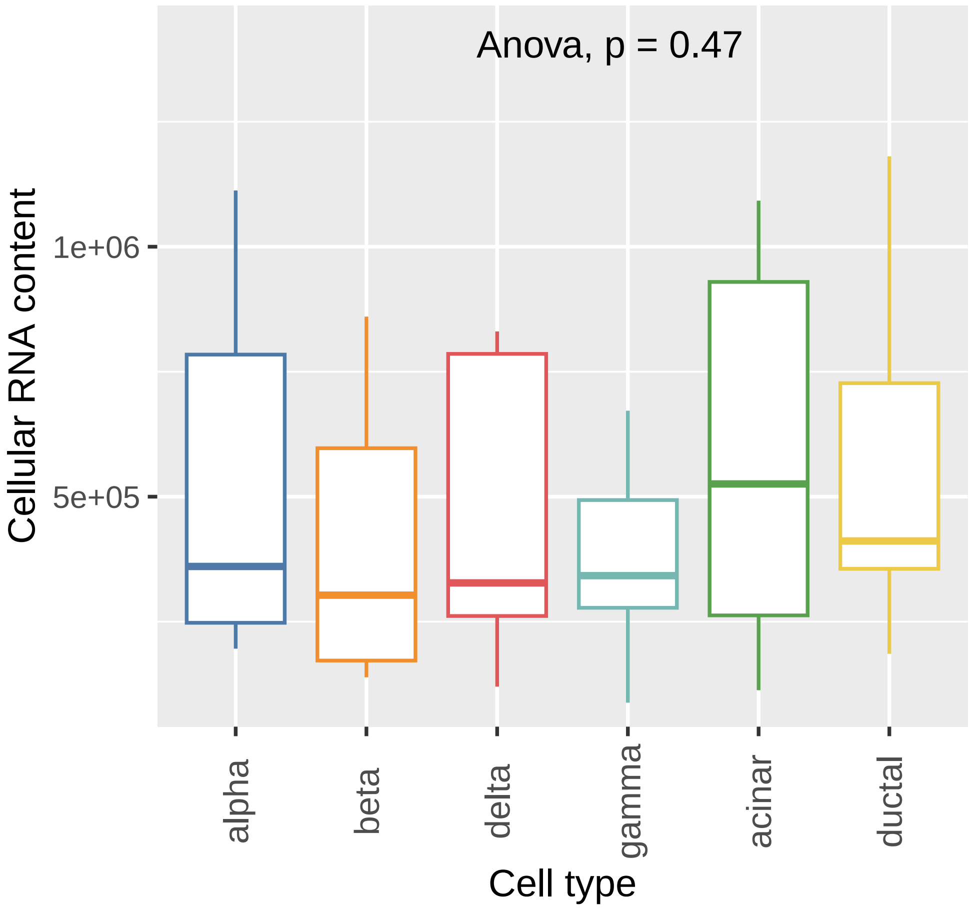

Figure S4: **Comparison of the cellular RNA content of six endocrine cell types in human pancreatic islet.** One-way ANOVA test was used to test whether mean cellular RNA contents of five immune cell types are statistically different.

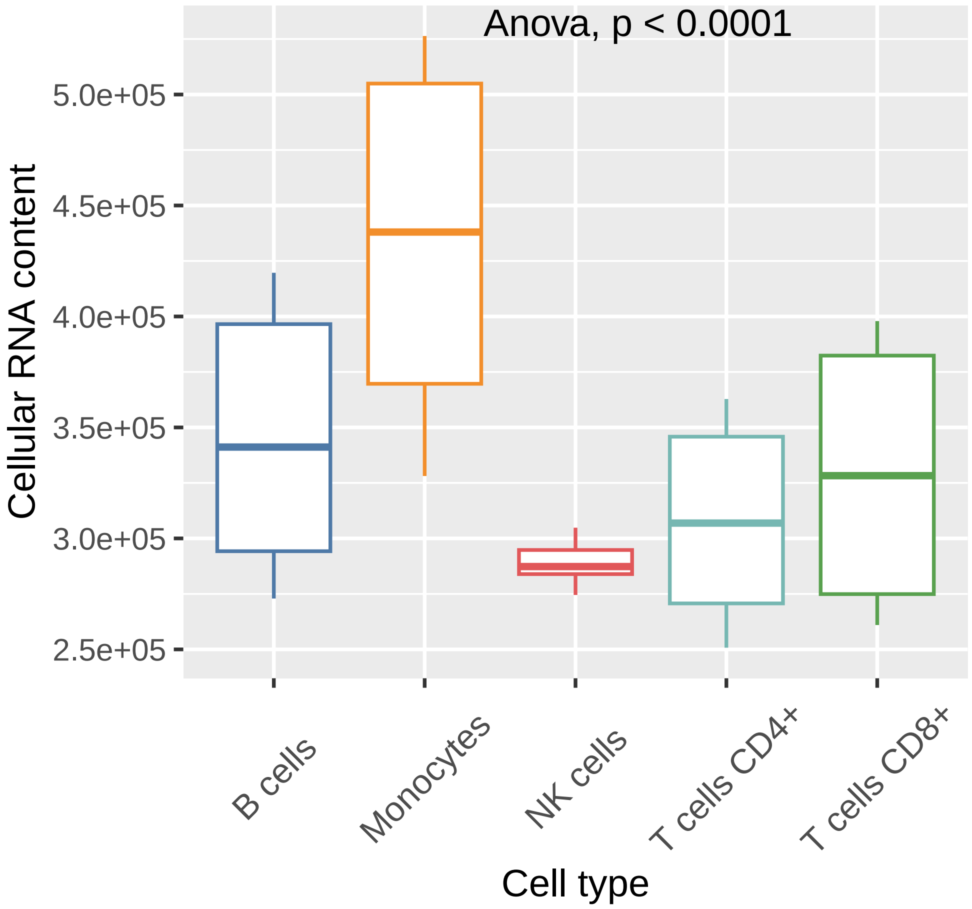

Figure S5: **Comparison of the cellular RNA content of five immune cell types in human PBMC.** One-way ANOVA test was used to test whether mean cellular RNA contents of five immune cell types are statistically different.

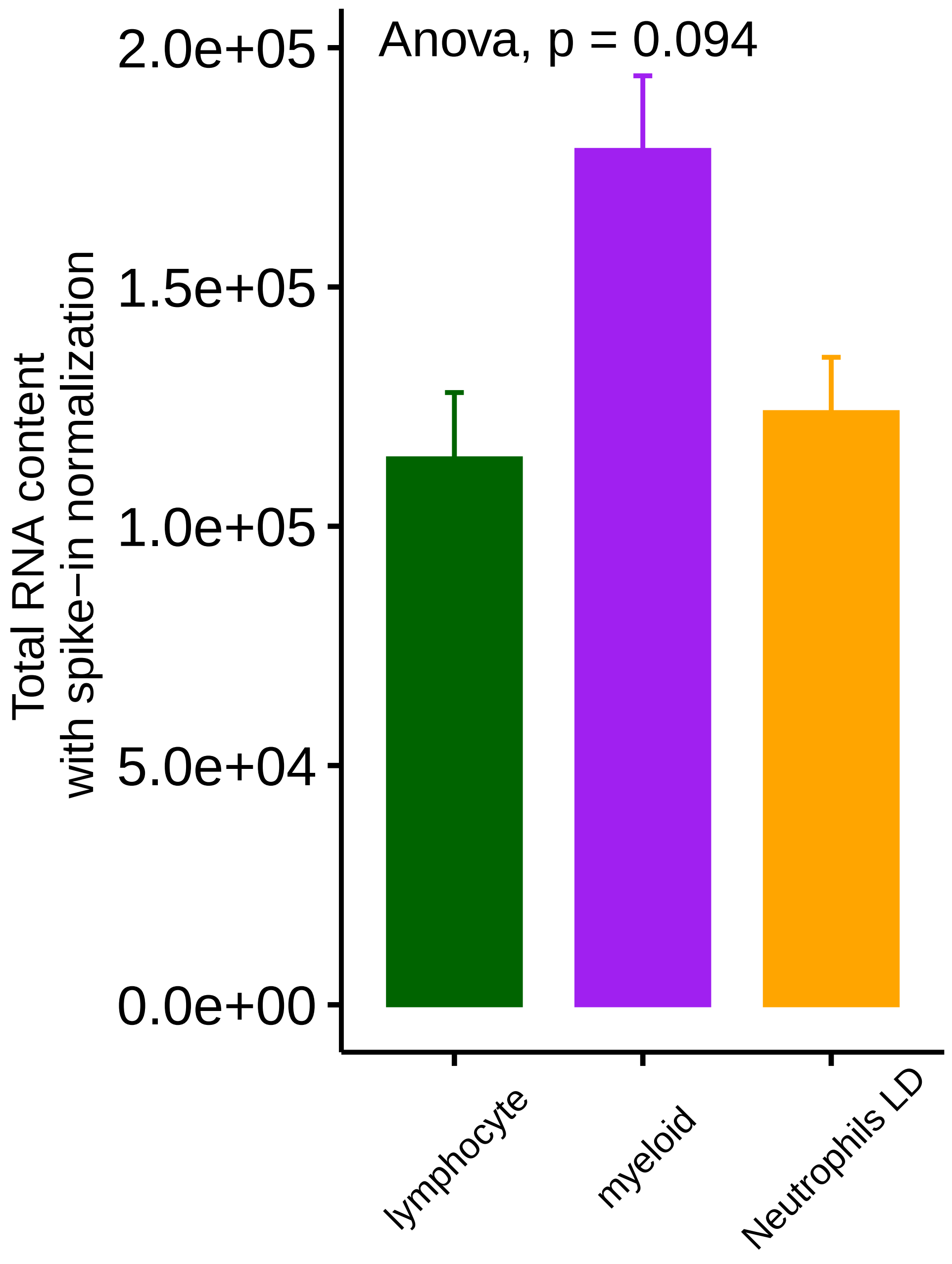

Figure S6: **Comparison of the estimated cell sizes of three large immune cell groups.** One-way ANOVA test was used to test whether the estimated cell sizes of three immune cell groups are statistically different.

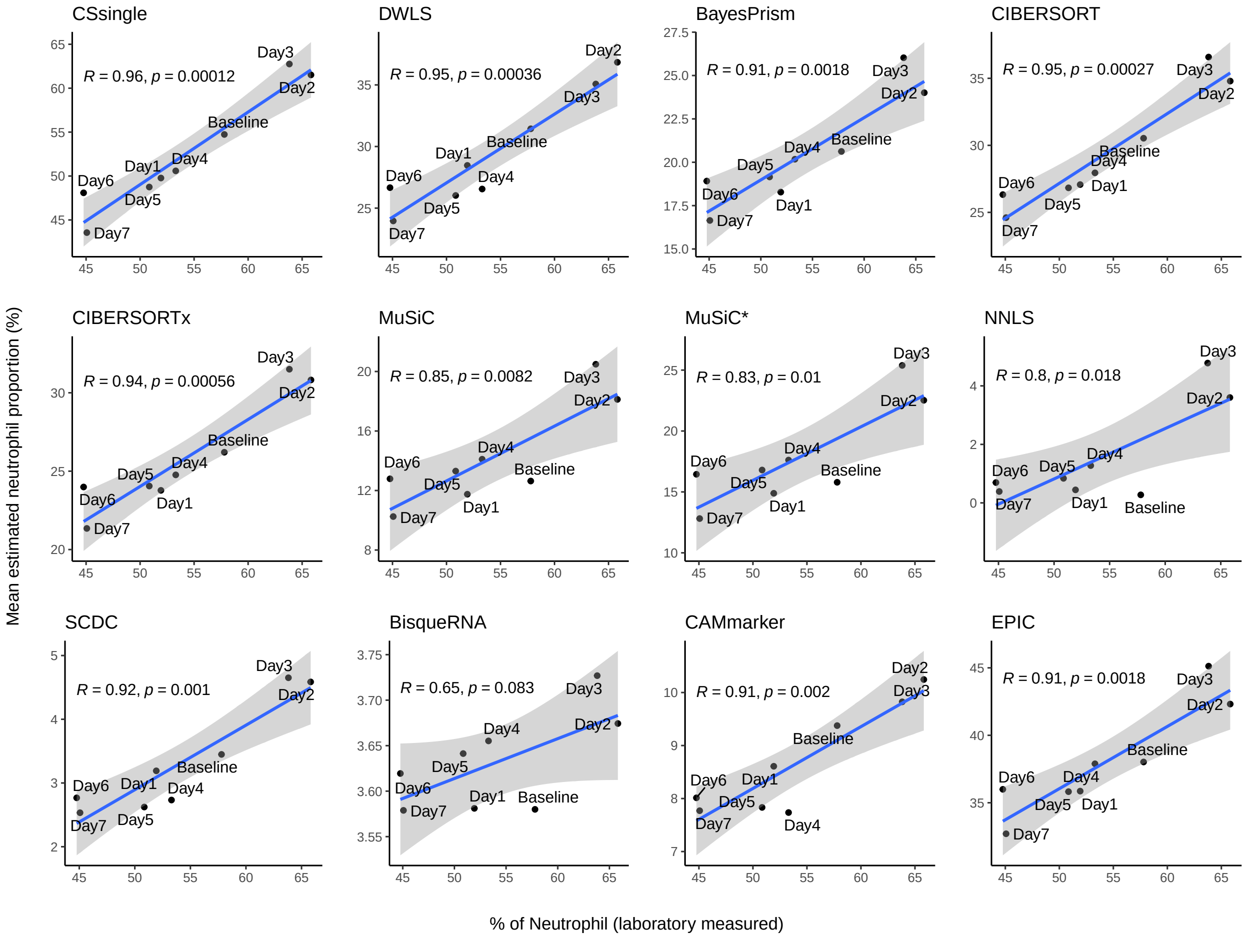

Figure S7: **Correlation between laboratory measured and estimated proportions of neutrophil in SI group of influenza H3N2.** Data points are labeled by the day post inoculation. Baseline represents prior to inoculation and day 1 represents the day of inoculation. Reported 'R' corresponds to Pearson's correlation and p-values indicate the significance of these correlations.

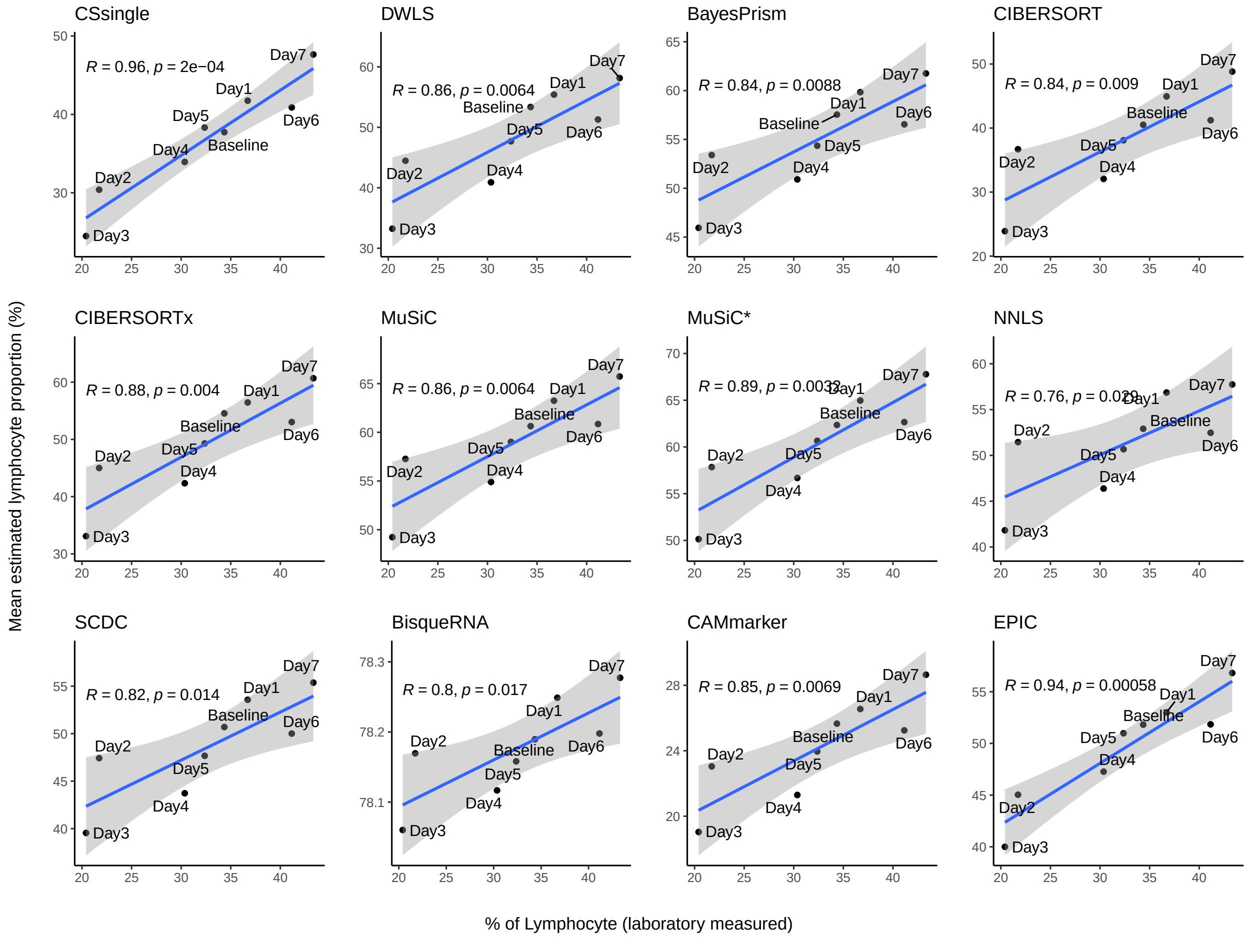

Figure S8: **Correlation between laboratory measured and estimated proportions of lymphocyte in SI group of influenza H3N2.** Data points are labeled by the day post inoculation. Baseline represents prior to inoculation and day 1 represents the day of inoculation. Reported 'R' corresponds to Pearson's correlation and p-values indicate the significance of these correlations.

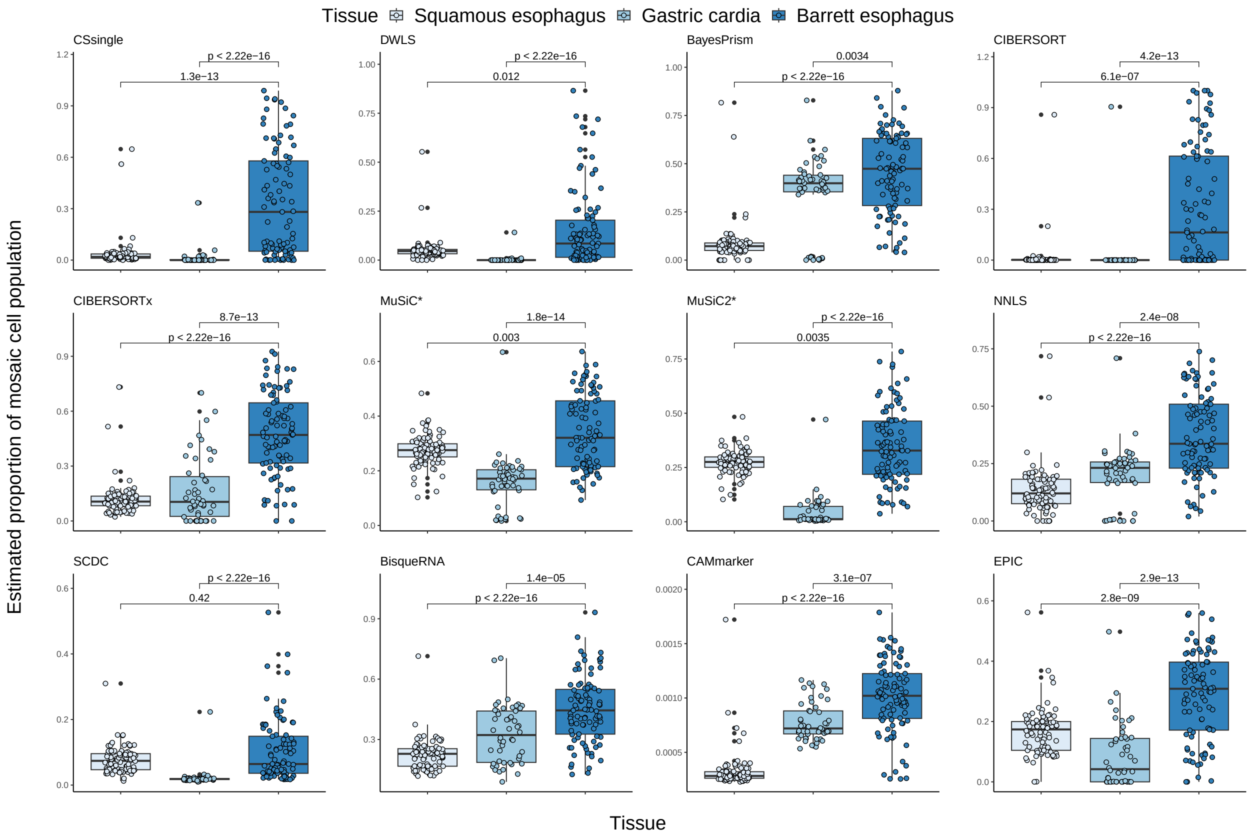

Figure S9: **Comparison of the estimated proportions of mosaic cell population in NE, NGC, and BE.** The p-values indicate the significance of the one-sided Wilcoxon-test. A p-value less than 0.05 is considered statistically significant, indicating the mosaic cell population is significantly increased in proportion in BE compared with the proportions in NE or NGC. Data are represented as boxplots (center line, median; box limits, upper and lower quartiles; whiskers, maximum and minimum values).

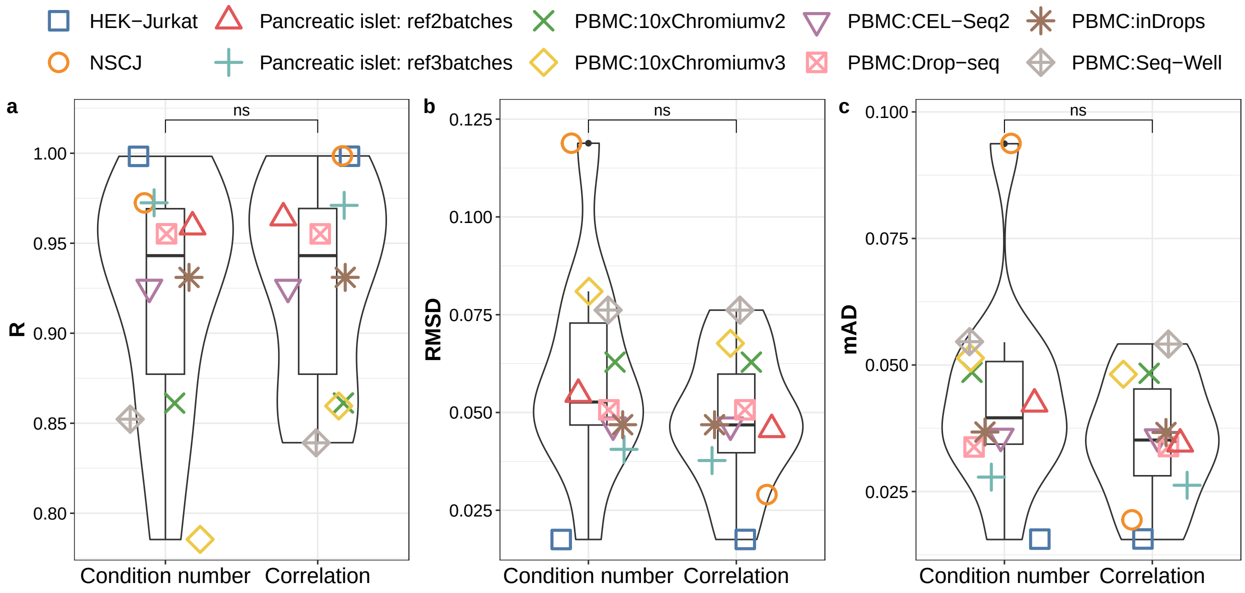

Figure S10: **Comparisons of the deconvolution results by integrating CSsingle with the signature matrix generated by different strategies. a,b,c** The evaluation plot depicting Pearson's correlation (**a**), root mean square deviation (**b**) and mean absolute deviation (**c**). Multiple signature matrices (six in total) were created by varying the number of marker genes from 50 to 300 with a step of 50 for each cell type. Ten data sets were denoted by different shapes and colors. Data are represented as boxplots (center line, median; box limits, upper and lower quartiles; whiskers, maximum and minimum values).

Table S1: Decomposition benchmark in human pancreatic islet tissue in terms of root mean square deviation (RMSD), mean absolute deviation (mAD) and Pearson's correlation (R).

Method	RMSD	mAD	R
CSsingle	0.046	0.035	0.964
DWLS	0.071	0.052	0.952
BayesPrism	0.066	0.051	0.938
CIBERSORT	0.089	0.067	0.878
CIBERSORTx	0.125	0.084	0.755
MuSiC	0.112	0.090	0.855
MuSiC2	0.120	0.094	0.844
NNLS	0.141	0.105	0.793
SCDC	0.178	0.120	0.583
BisqueRNA	0.122	0.082	0.798
CAMmarker	0.145	0.108	0.799
EPIC	0.189	0.137	0.522

Table S2: Performance of CSsingle and other methods when the single-cell reference data contains extra cell types. The proportions of cell type in the bulk data are shown in parentheses. 'others' include six rare cell types: activated stellate, quiescent stellate, schwann, macrophage, mast, and T cell.

**alpha(0.447)**	RMSD	mAD	R	**beta(0.137)**	RMSD	mAD	R	**delta(0.062)**	RMSD	mAD	R
CSsingle	0.05	0.04	0.95	CSsingle	0.03	0.03	0.90	CSsingle	0.04	0.03	0.79
DWLS	0.35	0.33	0.73	DWLS	0.12	0.11	0.90	DWLS	0.05	0.04	0.59
BayesPrism	0.07	0.06	0.94	BayesPrism	0.08	0.06	0.68	BayesPrism	0.04	0.03	0.55
CIBERSORT	0.20	0.17	0.82	CIBERSORT	0.09	0.08	0.51	CIBERSORT	0.02	0.02	0.96
CIBERSORTx	0.13	0.12	0.82	CIBERSORTx	0.07	0.06	0.63	CIBERSORTx	0.01	0.01	0.96
MuSiC	0.43	0.38	0.09	MuSiC	0.14	0.13	0.31	MuSiC	0.07	0.06	0.09
MuSiC2	0.43	0.38	0.10	MuSiC2	0.14	0.13	0.31	MuSiC2	0.07	0.06	0.09
NNLS	0.37	0.30	-0.06	NNLS	0.12	0.09	0.38	NNLS	0.06	0.05	0.17
SCDC	0.44	0.40	0.10	SCDC	0.14	0.12	0.26	SCDC	0.07	0.06	0.11
BisqueRNA	0.08	0.07	0.86	BisqueRNA	0.21	0.19	0.58	BisqueRNA	0.03	0.02	0.91
CAMmarker	0.17	0.15	0.91	CAMmarker	0.09	0.08	0.75	CAMmarker	0.07	0.06	0.62
EPIC	0.11	0.10	0.79	EPIC	0.14	0.12	0.49	EPIC	0.07	0.06	0.40
**gamma(0.092)**	RMSD	mAD	R	**acinar(0.084)**	RMSD	mAD	R	**ductal(0.177)**	RMSD	mAD	R
CSsingle	0.04	0.03	0.88	CSsingle	0.06	0.04	0.97	CSsingle	0.06	0.05	0.93
DWLS	0.08	0.06	0.90	DWLS	0.06	0.05	0.79	DWLS	0.34	0.31	0.83
BayesPrism	0.07	0.06	0.94	BayesPrism	0.05	0.04	0.95	BayesPrism	0.09	0.07	0.92
CIBERSORT	0.03	0.03	0.95	CIBERSORT	0.06	0.05	0.96	CIBERSORT	0.06	0.04	0.92
CIBERSORTx	0.04	0.03	0.94	CIBERSORTx	0.15	0.09	0.88	CIBERSORTx	0.07	0.06	0.89
MuSiC	0.11	0.08	0.35	MuSiC	0.12	0.08	0.80	MuSiC	0.21	0.17	0.65
MuSiC2	0.11	0.08	0.35	MuSiC2	0.12	0.08	0.80	MuSiC2	0.22	0.17	0.62
NNLS	0.08	0.06	0.60	NNLS	0.14	0.09	0.95	NNLS	0.15	0.12	0.79
SCDC	0.12	0.09	0.31	SCDC	0.12	0.08	0.86	SCDC	0.23	0.18	0.78
BisqueRNA	0.06	0.05	0.92	BisqueRNA	0.06	0.05	0.95	BisqueRNA	0.15	0.12	0.78
CAMmarker	0.12	0.09	0.86	CAMmarker	0.09	0.09	0.98	CAMmarker	0.23	0.17	0.97
EPIC	0.14	0.12	0.88	EPIC	0.09	0.06	0.96	EPIC	0.20	0.16	0.70
**epsilon(0)**	RMSD	mAD		**endothelial(0)**	RMSD	mAD		**others(0)**	RMSD	mAD	
CSsingle	0.01	0.01		CSsingle	0.00	0.00		CSsingle	0.00	0.00	
DWLS	0.29	0.24		DWLS	0.01	0.01		DWLS	0.00	0.00	
BayesPrism	0.03	0.02		BayesPrism	0.00	0.00		BayesPrism	0.03	0.02	
CIBERSORT	0.00	0.00		CIBERSORT	0.00	0.00		CIBERSORT	0.00	0.00	
CIBERSORTx	0.01	0.01		CIBERSORTx	0.02	0.02		CIBERSORTx	0.04	0.04	
MuSiC	0.64	0.59		MuSiC	0.32	0.27		MuSiC	0.00	0.00	
MuSiC2	0.65	0.60		MuSiC2	0.32	0.26		MuSiC2	0.00	0.00	
NNLS	0.25	0.11		NNLS	0.56	0.35		NNLS	0.00	0.00	
SCDC	0.76	0.69		SCDC	0.32	0.20		SCDC	0.00	0.00	
BisqueRNA	0.00	0.00		BisqueRNA	0.06	0.04		BisqueRNA	0.05	0.04	
CAMmarker	0.00	0.00		CAMmarker	0.00	0.00		CAMmarker	0.00	0.00	
EPIC	0.00	0.00		EPIC	0.08	0.05		EPIC	0.01	0.00	

Table S3: Performance of CSsingle and other methods in the human PBMC. The proportions of cell type in the bulk data are shown in parentheses. The RMSE, mAD and R values were averaged across six different scRNA-seq methods. The smallest RMSD and mAD, and the largest R values for each cell type are highlighted in bold.

**B cells(0.152)**	RMSD	mAD	R	**Monocytes(0.152)**	RMSD	mAD	R
CSsingle	**0.03**	**0.02**	**0.99**	CSsingle	**0.02**	**0.02**	0.95
DWLS	0.04	0.04	**0.99**	DWLS	0.1	0.09	0.96
BayesPrism	0.06	0.06	**0.99**	BayesPrism	0.14	0.13	0.96
CIBERSORT	0.09	0.08	0.95	CIBERSORT	0.07	0.06	0.87
CIBERSORTx	0.07	0.07	0.95	CIBERSORTx	0.08	0.07	0.92
MuSiC	0.14	0.13	0.61	MuSiC	0.1	0.09	0.83
NNLS	0.14	0.13	-0.19	NNLS	0.22	0.2	0.69
SCDC	0.13	0.12	0.64	SCDC	0.08	0.07	0.8
BisqueRNA	0.06	0.05	0.56	BisqueRNA	0.09	0.09	0.05
CAMmarker	0.06	0.06	0.99	CAMmarker	0.03	0.03	**0.97**
EPIC	0.16	0.13	0.58	EPIC	0.46	0.4	0.54
**T cells CD4+(0.220)**	RMSD	mAD	R	**NK cells(0.092)**	RMSD	mAD	R
CSsingle	**0.05**	**0.04**	0.37	CSsingle	0.08	0.06	0.91
DWLS	0.12	0.11	-0.39	DWLS	**0.05**	**0.04**	0.96
BayesPrism	0.16	0.15	0.32	BayesPrism	0.28	0.26	**0.99**
CIBERSORT	0.12	0.11	-0.47	CIBERSORT	0.1	0.08	0.77
CIBERSORTx	0.16	0.13	-0.56	CIBERSORTx	**0.05**	**0.04**	0.96
MuSiC	0.17	0.17	-0.04	MuSiC	0.38	0.37	0.33
NNLS	0.27	0.26	0.02	NNLS	0.22	0.2	0.55
SCDC	0.15	0.15	0.24	SCDC	0.42	0.41	0.58
BisqueRNA	0.08	0.07	**0.59**	BisqueRNA	0.07	0.06	0.94
CAMmarker	0.12	0.12	0.52	CAMmarker	0.08	0.06	0.98
EPIC	0.21	0.21	-0.03	EPIC	0.22	0.19	0.11
**T cells CD8+(0.384)**	RMSD	mAD	R				
CSsingle	**0.08**	**0.06**	0.84				
DWLS	0.13	0.12	0.9				
BayesPrism	0.19	0.17	0.91				
CIBERSORT	0.13	0.11	**0.96**				
CIBERSORTx	0.14	0.12	**0.96**				
MuSiC	0.3	0.28	-0.1				
NNLS	0.32	0.29	-0.61				
SCDC	0.24	0.22	-0.08				
BisqueRNA	0.11	0.09	0.53				
CAMmarker	0.14	0.11	0.79				
EPIC	0.35	0.32	0.04				

Table S4: Runtime efficiency and deconvolution accuracy for different step sizes used in the signature matrix construction. Multiple signature matrices were created by varying the number of marker gens from 50 to 300 with step 50 (six in total) or 1 (251 in total) for each cell type. All datasets were run on a 2.59 GHz Intel Xeon Processor with 256 GB of RAM and 32 cores. R's time module was used to obtain runtime measurements in seconds.

	step size = 50					step size = 1				
Datasets (# samples)	RMSD	mAD	R	Optimal #	Runtime(s)	RMSD	mAD	R	Optimal #	Runtime(s)
Pancreatic islet:ref2batches (10)	0.05	0.03	0.96	50	0.63	0.04	0.03	0.97	53	20.62
PBMC:10xChromiumv2 (200)	0.06	0.05	0.86	100	5.18	0.06	0.05	0.87	71	213.87
PBMC:10xChromiumv3 (200)	0.07	0.05	0.86	100	5.1	0.07	0.05	0.84	78	205.66
PBMC:CEL-Seq2 (200)	0.05	0.04	0.93	50	5.55	0.05	0.04	0.93	50	232.77
PBMC:Drop-seq (200)	0.05	0.03	0.96	50	5.03	0.05	0.04	0.93	71	200.84
PBMC:inDrops (200)	0.05	0.04	0.93	100	4.8	0.06	0.04	0.9	74	196.87
PBMC:Seq-Well (200)	0.08	0.05	0.84	100	4.18	0.08	0.06	0.85	73	169.81
